# Advancements in FR4 dielectric analysis: Free space approach and measurement validation

**DOI:** 10.1371/journal.pone.0305614

**Published:** 2024-09-12

**Authors:** Syed Zeeshan Ali, Kamran Ahsan, Danish ul Khairi, Wadee Alhalabi, Muhammad Shahid Anwar

**Affiliations:** 1 Department of Computer Science, Federal Urdu University Arts and Science Technology, Karachi, Sindh, Pakistan; 2 Faculty of Engineering and Computer Science, Millennium Institute of Technology and Entrepreneurship, Karachi, Pakistan; 3 Immersive Virtual Reality Research Group, Department of Computer Science, King Abdulaziz University, Jeddah, Saudi Arabia; 4 Department of AI and Software, Gachon University, Seongnam-si, South Korea; Chandigarh University, INDIA

## Abstract

In this study, the free space approach is utilized to calculate the relative permittivity of FR4 by utilizing the Nicholson-Ross-Weir Conversion. By examining the scattering characteristics, the free space technique offers a practical tool for describing dielectric materials. The simulations were run on CST-2019, and the frequency range of 8.5 GHz to 11.5 GHz was chosen. Experimental measurements were carried out utilizing a Vector Network Analyzer, To further reduce outside influences and assure accurate measurements in a controlled setting, an anechoic chamber was used. The outcomes of the simulations and actual measurements show the significance of the Nicholson- Ross-Weir Conversion and free space approach in calculating the relative permittivity of FR4. The correctness and dependability of the suggested technique are confirmed by the good agreement between the simulated and measured outcomes. This study makes a contribution to the field of electromagnetic characterization and offers a useful method for figuring out FR4’s dielectric characteristics. The results of this study have substantial effects on PCB design and optimization as well as other high-frequency electronic devices that operate in the frequency band under consideration.

## Introduction

The need for high-frequency electronics has increased significantly in the last few years, which has sparked the creation of novel materials specifically designed to be integrated into electronic circuits [1–3]. The construction of printed circuit boards (PCBs) uses a variety of materials, but FR4 (Flame Retardant 4) is very popular because of its superior electrical characteristics like variety of dielectric values, Loss tangent, high break down voltage, low cost, and simplicity of production [4–6]. For high-frequency electrical devices to operate as efficiently as possible, the dielectric characteristics of FR4 must be accurately characterized [7, 8]. The development of electronic and flexible circuits that operate at microwave frequencies is also critically dependent on the microwave characteristics of materials. Flex circuits are often used in computer peripherals, mobile phones, cameras, calculator, printers, and LCD manufacture [9–12]. In the field of microelectronics and thin-film technology, the research team aimed to investigate the properties of a specialized film affixed to a substrate. This film, a critical component in various electronic devices, was subjected to extensive analysis. A few micrometres of thickness are visible in the film that was placed on top of the substrate [13].

Knowledge of the electromagnetic properties of materials is essential for both manufacturers and users [14]. Due to the significance of the frequency range, several measuring techniques are employed [15]. Traditional techniques for determining a material’s dielectric characteristics, nevertheless, have complexity, frequency range, and accuracy restrictions.

Maxwell’s equations illustrate the interaction of electromagnetic radiation with materials, where material properties such as permittivity and permeability depend on frequency [16, 17]. Under-tested transmission or reflection techniques are used to compute the complex permittivity and permeability of materials such as isotropic ones from sample measurements. For nonmagnetic samples, measuring just one coefficient either reflection or transmission is sufficient to determine the complex permittivity value [18]. It is important that the sample size measured at microwave frequencies is usually bigger to the wavelength [19]. Maxwell’s equations’ numerical solutions must be used to determine how the measured and calculated values are related to one another.

The characterization of dielectric materials is crucial in various fields. The free-space method offers a valuable solution by enabling the efficient and non-destructive evaluation of both their electrical and, in some cases, magnetic properties. This technique proves particularly useful for materials like FR4. The free-space approach allows for a straightforward characterization process without damaging the sample [20]. This approach provides simplicity, versatility, and a wide frequency range of operation as compared to traditional approaches like coaxial line resonators and waveguide techniques. The development in FR4 dielectric analysis makes a significant contribution to the field of electromagnetic characterization and provides a useful method for precisely determining the dielectric properties of FR4 as well others materials. The use of the free space technique and measurement validation strategies has received the majority of attention in the development of FR4 dielectric evaluation. This has significant implications for PCB design, optimization, and the creation of high-frequency electronic devices operating within the target frequency band.

In the field of electromagnetic characterization, the Nicholson-Ross-Weir (NRW) approach is crucial. Design and optimization of high-frequency electronic devices depend on its unparalleled precision in measuring the relative permittivity of materials such as FR4. This is especially important for improving simulation accuracy and antenna technology. To illustrate the NRW method’s versatility and its profound impact on antenna technology, we reference seminal studies where it played a pivotal role in optimizing microstrip patch antennas using omega-shaped metamaterial lenses [21] and enhancing directivity with inverse refraction metamaterials [22]. From the s-parameters, The algorithm Nicholson-Ross-Weir (NRW), directly calculates the permittivity and permeability [23–25]. In order to get important insights into the material’s dielectric properties, this method entails analyzing the electromagnetic wave scattering characteristics in a free space environment. It is necessary to measure all four (S11, S21, S12, S22) or a pair (S11, S21) of the characteristics of the material under test in order to evaluate the reflection coefficient and transmission coefficient [26, 27]. The accuracy and reliability of the recommended approach are confirmed by the high agreement between simulated and measured outcomes.

Controlled laboratory settings are frequently necessary for the characterization of electromagnetic waves. Since anechoic chambers can reduce undesired reflections and scattering, we used one in this work, which is a usual setup for these kinds of experiments. Reverberation chambers (RCs) provide a cost-effective alternative to anechoic chambers (ACs) for electromagnetic compatibility (EMC) testing. Ensuring uniform electric field distribution defines the working volume (WV), traditionally determined through time-consuming adjustments. Reverberation chambers, however, provide an alternate strategy that should be acknowledged, especially when working with low intended signal levels [28].

With the free space technique, this work aims to determine the material permittivity of FR4, utilizing a pair of spot-focusing lenses. The existing literature highlights that the size of the sample under examination is typically large, the cost of the lenses used is relatively high, and accuracy errors are caused by low-loss materials and interference of background signals from transmission/reflections. However, in this study, a cost-effective solution is proposed by utilizing an adjustable pair of lenses, that can adjust the beam-width of radiation by modifying the feed and lens position, improving the accuracy of the measurements. Furthermore, the cost-effectiveness and simplicity of the lens design make it an attractive solution for researchers and practitioners working in the field of material characterization and permittivity determination.

Overall, this work advances the state of the art by presenting a novel method for overcoming problems with sample size, lens price, accuracy mistakes, and background signals. This work makes a significant addition to the study of material permittivity determination due to the use of spot-focusing lenses and their distinct design features. The first section provides a concise overview of the investigation. The second section details the experimental arrangement and its illustration. Lastly, the third section showcases the test procedures and confirms the outcomes.

## Design and optimization of obsolete hardware configurations for enhanced hardware setup design

**Fig 1:**
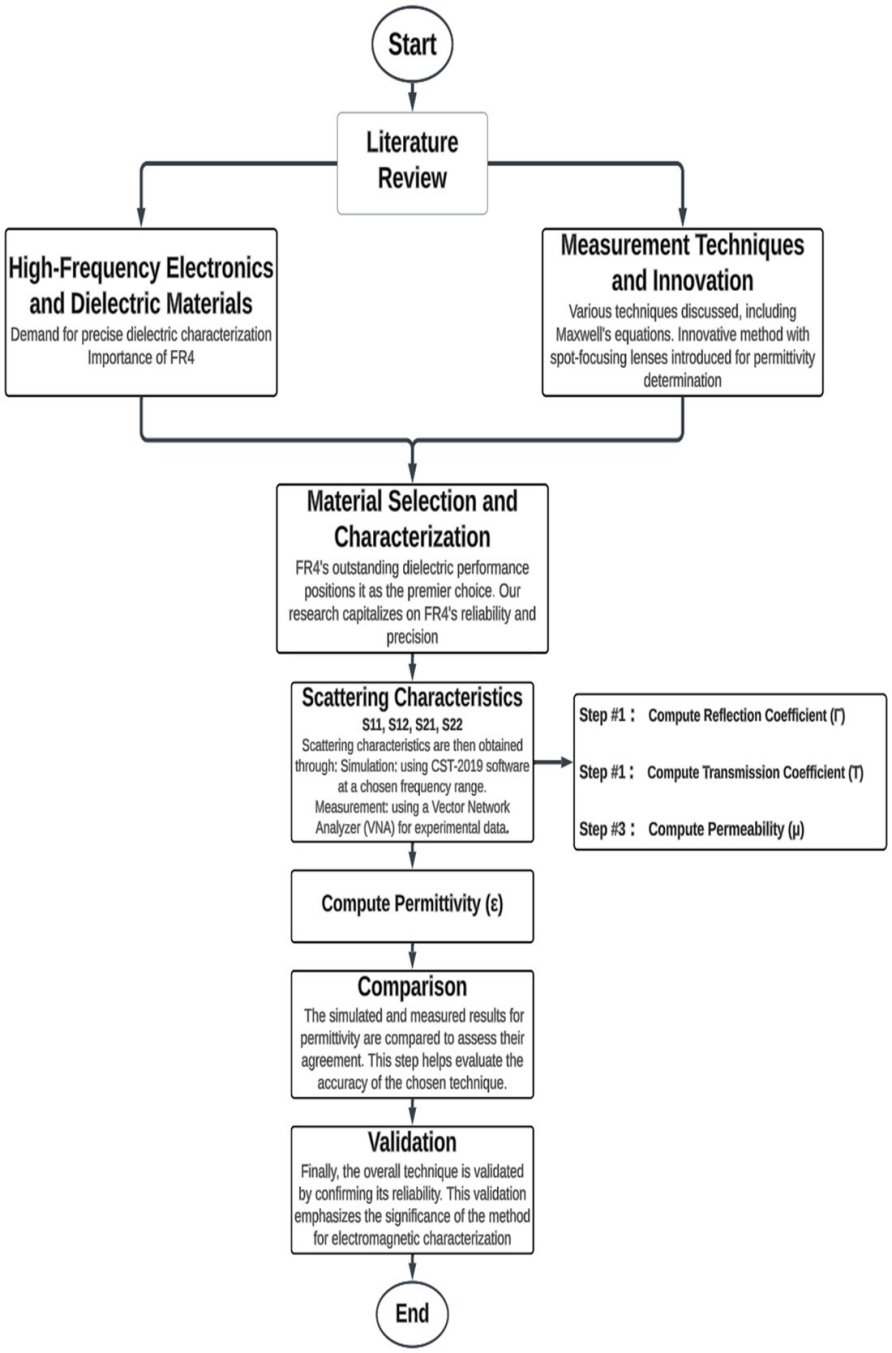
Flowchart of the overall experimental process. This figure illustrates the sequential steps involved in the research experiment.

Fig 1 presents a concise flowchart outlining the two-step experimental process employed to evaluate the effectiveness of the free space approach for calculating FR4 permittivity. This research involved the development and simulation of a sophisticated system utilizing lens and horn antennas. The primary objective of this meticulously designed setup was to precisely direct and focus energy towards the FR4 dielectric material, facilitating thorough investigation and accurate measurement. Although the outward appearance of this system may seem unassuming and basic, it was deliberately chosen as the initial platform for our research. This deliberate selection was driven by the necessity to establish a robust foundation and serve as a starting point for our comprehensive investigations. Despite its deceptively straightforward appearance, this system played a pivotal role in underpinning our research endeavors, empowering us to progressively refine and augment our methodologies as we advanced.

A pair of horn antennas with a directivity of 16.8 dBi and beam-width 27.50 degrees was chosen. This specific directivity and beam-width values were determined to be optimal for this study, taking into account the desired performance and functionality requirements. In order to enhance the directivity even further, a pair of lenses has been selected with specific dimensions. The lenses had a thickness (T) of 24.34 mm, an outer radius (R) of 74.34 mm, and a length (L) of 110 mm. Additionally, the focal length (f) of the lenses was set at 165.5 mm. These dimensions were chosen to optimize the focusing capabilities of the lenses. Utilizing the lenses after horn antenna the system exhibited a directivity of 21.9 dBi, indicating their ability to concentrate energy in a particular direction with increased gain. The beam-width of the lens antennas was determined to be 13.10^0^, defining the angular coverage of the focused energy.

**Fig 2:**
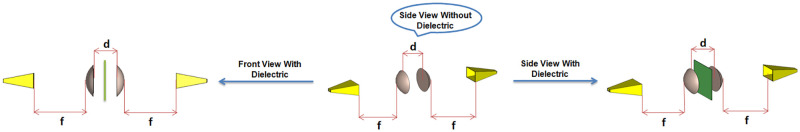
Combined view of the hardware setup for determining the dielectric constant value of a material: Dielectric-free perspective, front view with FR4 substrate, and perspective view with FR4 substrate.

CST software is used to optimize the transmission and reception of signals. The positioning of the dielectric lens antennas, a pair of horn antennas, and FR4 material was such that their centers were in the line of sight (LOS). This improved the design of the dielectric lens antennas. The setup was fully capable to utilize the free space method to perform precise measurements and analysis of the dielectric characteristics of FR4 as shown in Fig 2.

Focusing electromagnetic waves requires the focal length of the lens system (f). In order to achieve the desired interaction between the electromagnetic field and the sample, the dielectric sample’s distance (D) and the lens’ center-to-center spacing (d) were mentioned optimized. Table 1 summarizes the key optimized parameters obtained through simulations.

**Table 1:** Parameters for the lens system used in the characterization of a dielectric material.

Serial No	Parameter (mm)	Symbol	Value (mm)
1	Focal Length of Lens System	f	165.50
2	Center-to-Center Lens Spacing	d	75.50
3	Dielectric Sample-to-Lens Separation	D	37.75

**Fig 3:**
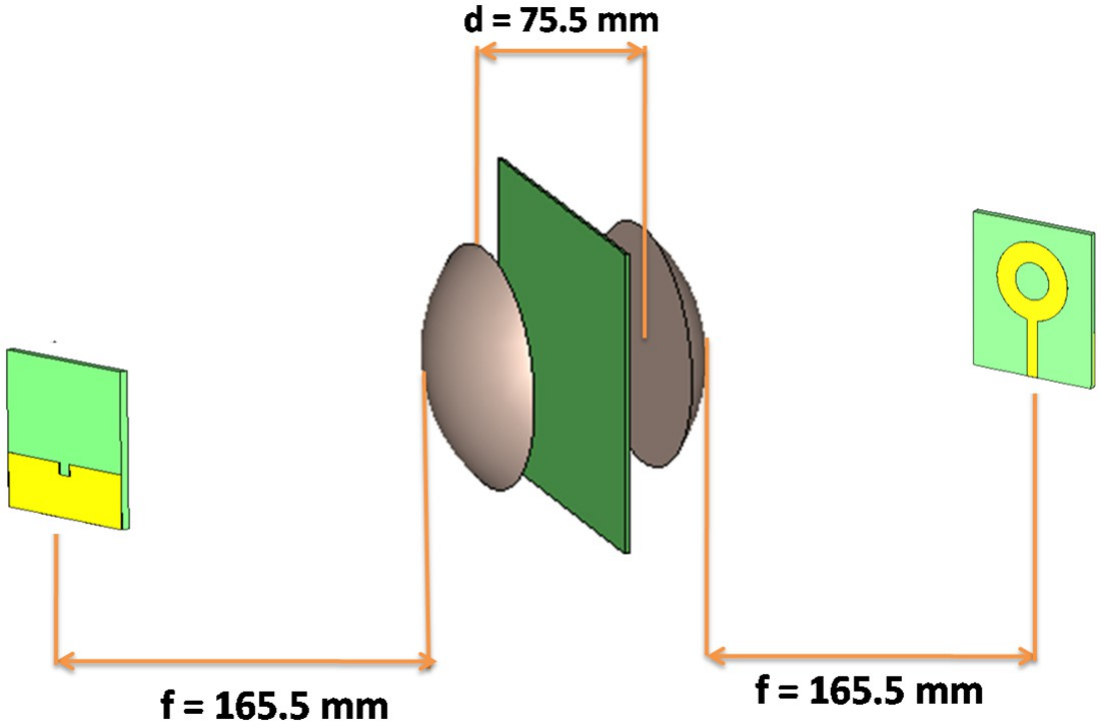
Design view of the complete hardware setup incorporating a patch antenna for precise determination of the dielectric constant value of a material.

The data illustrating the influence of lens design on directivity and beam focusing at various distances is presented in [S1 Table]. Circular ring patch antennas have taken the role of the conventional horn antennas in the analysis of the permittivity of FR4 materials. This cutting-edge method delivers notable characteristics, such as wider frequency coverage and improved impedance matching, allowing in-depth investigation of permittivity values. In Fig 3 the design view of the complete hardware setup incorporating a patch antenna has been shown.

## Experimental setup and demonstration

The experimental setup utilizes the following components, each playing a crucial role in the exploration and measurement of a material’s dielectric constant.

- A pair of Horn antennas of 16.8 dBi directivity and 27.5^0^ beam-widths.
- A pair of Lenses have dimensions of T = 24.34 mm, R = 74.34mm, L = 110mm, f = 165.5mm, and also have 21.9 dBi directivity and 13.1^0^ beam-width.
- A vector network Analyzer for generating S2p files.
- Two holding frames for lens and two holding frames for antennas.

Measurement of the sample’s dielectric Free space measurement, which often makes use of broadband frequency, is capable of being accurate even in harsh conditions and high temperatures. The testing material must be large and flat in order to meet the requirements of free space procedures. In our experiment, we positioned an FR4 material, and the size of the testing material in terms of wavelengths is determined by the ratio of the distance between the materials and the antenna in meters to the operating wavelength. Specifically, for the frequency range spanning from 9 GHz to 12 GHz, we find that the size of the testing material is roughly equivalent to 0.333 wavelengths. The vector network analyzer is linked to both of the two antennas, which are typically located in front of one another.

The VNA needs to be calibrated first, then the measurement can begin. A variety of calibration techniques, including the through-reflect-line (TRL), the through-reflect-match (TRM), and the line-reflect-line, can be utilized (LRL). The best calibration quality, though, can be produced through the LRL calibration technique. The line standard criteria can be obtained by separating the two antennas’ focal planes by about a quarter of a wavelength. A metal plate can be used to create the reflect standard by positioning it within the sample holder between the antennas.

After calibration, an empty sample holder’s s-parameters are determined by putting it halfway between the two antennas. The matarial under test (MUT) [29] is then placed on the sample holder between the antennas and the s-parameter measurement is performed again. Using the de-embedding function of the VNA, the influence of the sample holder can be canceled out and only the s-parameter of the MUT can be determined. The s-parameter for both, the reflection and transmission coefficients can be determined. Time domain gating should also be applied to ensure there are no multiple reflections in the sample itself, though appropriate thickness should be able to avoid this. S-parameter conversion methods detailed in [S1 File] were employed to measure material dielectric properties using a network analyzer.

Since the Nicholson-Ross-Weir transformation is being implemented, it is necessary to verify the correctness of the results using this algorithm. First, the dielectric value of the known material needs to be found. The FR4 material with a dielectric value of 4.7 was chosen. A Band of 10–12 GHz was selected on VNA. After obtaining S2p files, the files are processed on the MATLAB program. The complete MATLAB code has been presented in [S2 File]. The NRW method proposes an equation for determining the relative permittivity. This research paper adopts the proposed equation to accurately calculate the relative permittivity.

$$\begin{array}{c} \varepsilon_{r}=\frac{\lambda_{0}^{2}}{\mu_{r}}\left[\frac{1}{\lambda_{c}^{2}}-{\left(\frac{1}{2\pi L}\text{ln}\left(\frac{1}{T}\right)\right)}^{2}\right] \end{array}$$ (1)

L = material length.

*ε*_*r*_ = relative permittivity.

*μ*_*r*_ = relative permeability.

λ_*g*_ = wavelength in sample.

λ_0_ = cutoff wavelength

*π* = 3.14

Γ = propagation constant of material.

c = velocity of light.

f = frequency.

A real part (dielectric constant), which measures energy storage under an electric field, and an imaginary part (loss factor), which indicates energy dissipation, make up the complex dielectric permittivity $\left(\varepsilon_{r}^{*}\right)$. The ratio of these components is “tan *δ*”, often known as the loss tangent, tangent loss, dissipation factor, or loss factor. While the imaginary part of complex permittivity $\left(\varepsilon_{r}^{*}\right)$ is a crucial characteristic of materials. In Eq (1), ‘$\text{ln}(\frac{1}{T})$’ represents the imaginary component of permittivity, denoting the portion of permittivity that corresponds to the imaginary part of the complex dielectric constant. It’s important to note that this research primarily focuses on evaluating the real part of complex permittivity.

To determine the permittivity (dielectric constant), Eq (1) is employed [29], requiring the evaluation of the parameters mentioned in Eqs (2), (3), (4), (5) and (6), as follows.

$$\begin{array}{c} S_{11}=\frac{\Gamma (1-T^{2})}{\left(1-\Gamma^{2}T^{2}\right)} \end{array}$$ (2)

$$\begin{array}{c} S_{21}=\frac{T(1-\Gamma^{2})}{1-\Gamma^{2}T^{2}} \end{array}$$ (3)

The parameters *S*_11_ and *S*_21_ are obtained directly from the network analyzer The reflection coefficient can be deduced as

$$\Gamma =X\sqrt[{}]{X^{2}-1}$$ (4)

where |Γ1| < 1 is required for finding the correct root and in terms of s-parameter can be optimized as

$$\begin{array}{c} X=\frac{S_{11}^{2}+S_{21}^{2}+1}{2S_{11}} \end{array}$$ (5)

The transmission coefficient can be written as:

$$\begin{array}{c} T=\frac{S_{11}+S_{21}-\Gamma }{1-(S_{11}+S_{21})\Gamma } \end{array}$$ (6)

**Fig 4:**
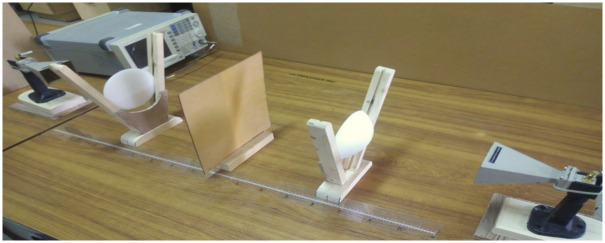
Practical setup of a microwave system for measuring the dielectric constant of FR4 in free space. The system includes a microwave source, lens, and receiving antenna.

A well-documented FR4 material with a standard dielectric value of 4.7 was chosen. This initial step confirmed that the actual dielectric constant of the FR4 material aligns with the reported standard value. However, the output average value of dielectric for the entire bandwidth was 1.23 and certainly, it is inaccurate. There are many factors that can affect the accuracy, in which reflections of energy from the surrounding is a dominant factor. To minimize the reflection from the surrounding we need a highly directive beam, so we placed our designed dielectric lens antenna after the feed antenna as shown below in Fig 4.

After using the pair of lenses, the permittivity values for the whole band are extremely close to the true value of FR4, which is discussed in the results section.

Advanced hardware is being developed to determine the dielectric constant values of materials. This hardware includes a circular plate connected to a stepper motor that can rotate the object being tested by 360^0^. There are three integrated programming alternatives available:

1. **Continuous Rotation:** By pressing the button once, the object rotates in steps of 1^0^ continuously.
2. **Step Size of 5^0^:** Each time the button is pressed, the object rotates by a step size of 5^0^.
3. **Step Size of 30^0^:** Each time the button is pressed, the object rotates by a step size of 30^0^.

The rotating plate is surrounded by an 18-inch-diameter acrylic cylinder on which the transmitter and receiver antennas are mounted, allowing them to rotate 360^0^ around the material under test. The precise horizontal and vertical movement of the antennas can also be performed using a scale.

**Fig 5:**
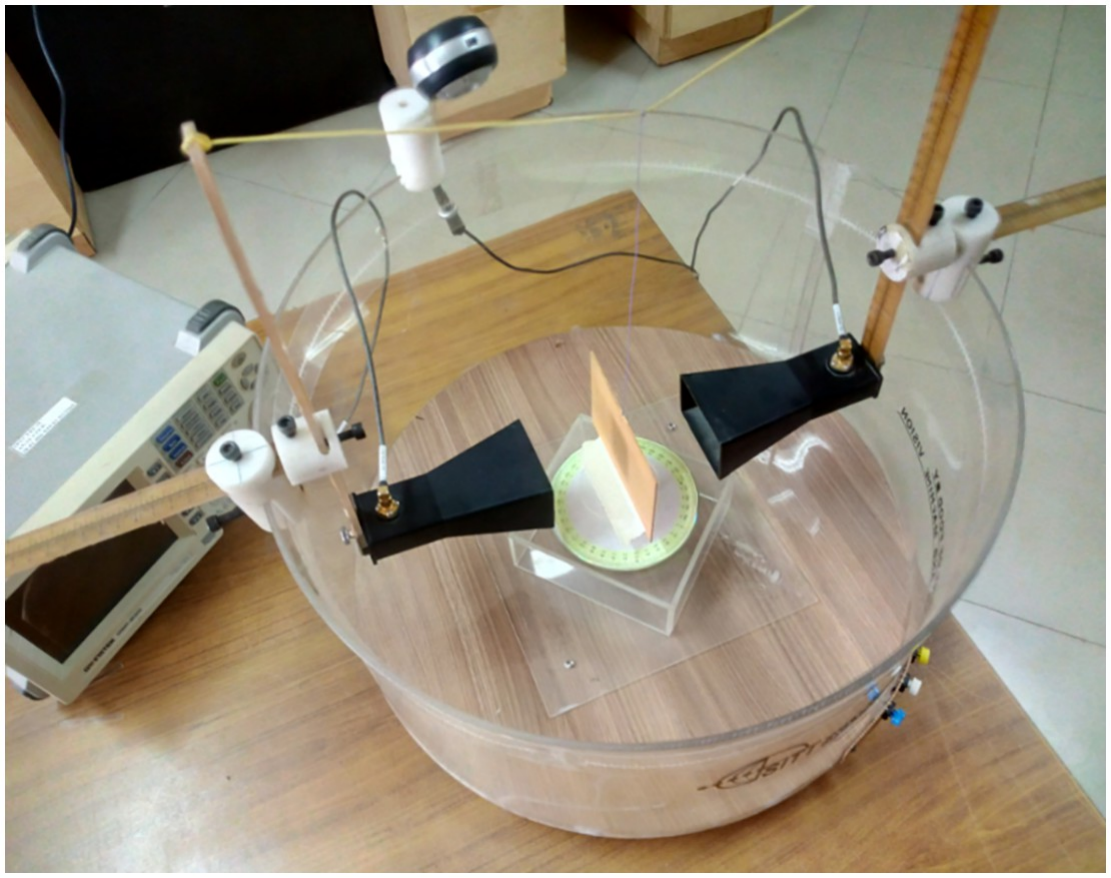
Advance integrated setup with horn antenna and FR4 for precise dielectric value determination.

To assess this advanced and state-of-the-art hardware setup performance and accuracy in calculating dielectric properties, same horn antennas are used in this system as shown in Fig 5. However, the results obtained for the dielectric value of FR4 are inaccurate across the entire frequency band. This inaccuracy is primarily attributed to reflections of energy from the surrounding environment, which is a dominant factor affecting the accuracy of the measurements.

**Fig 6:**
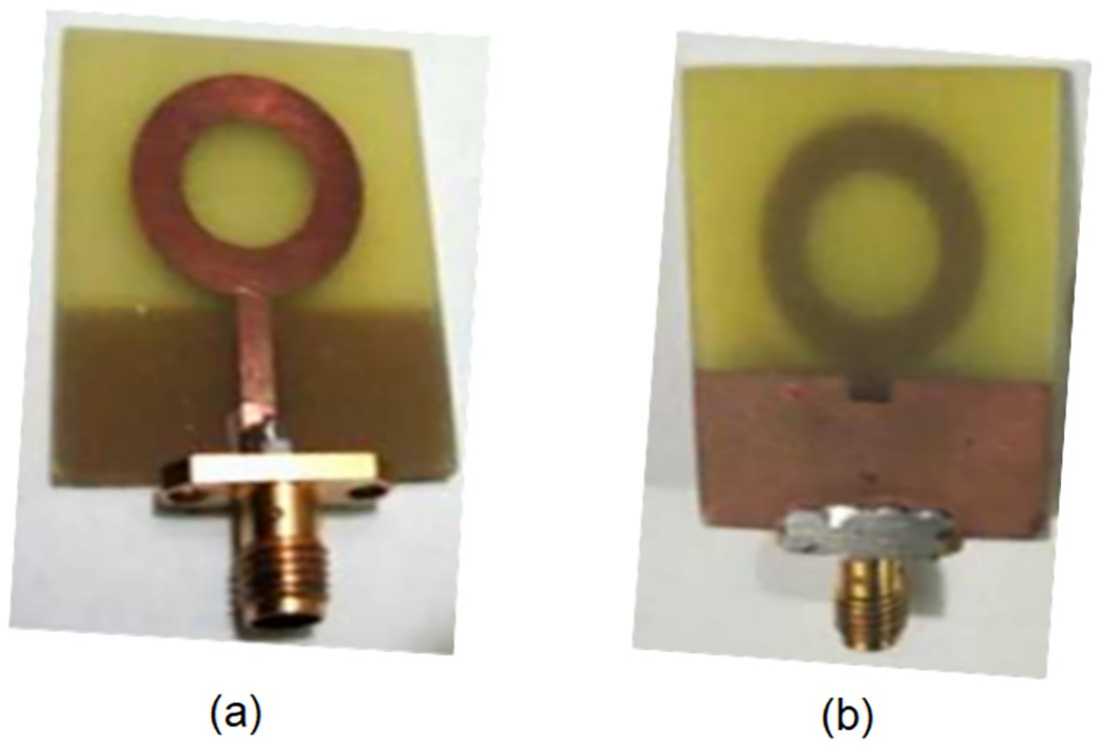
(a) Front view and (b) Back view of the truncated ground plane ring type patch antenna.

To overcome the size constraints associated with using horn antennas for beamforming, an alternative approach was implemented. Patch antennas with lenses were employed instead to achieve similar beamforming capabilities. The experimental setup involved the utilization of a circular ring patch antenna, featuring a truncated ground plane, on an FR-4 substrate with dimensions of 33mm × 28mm × 1.57mm. They exhibit a wide operational bandwidth spanning from 2.75 GHz to 32.035 GHz, while maintaining a VSWR (Voltage Standing Wave Ratio) below 2 [30]. as shown in Fig 6.

**Fig 7:**
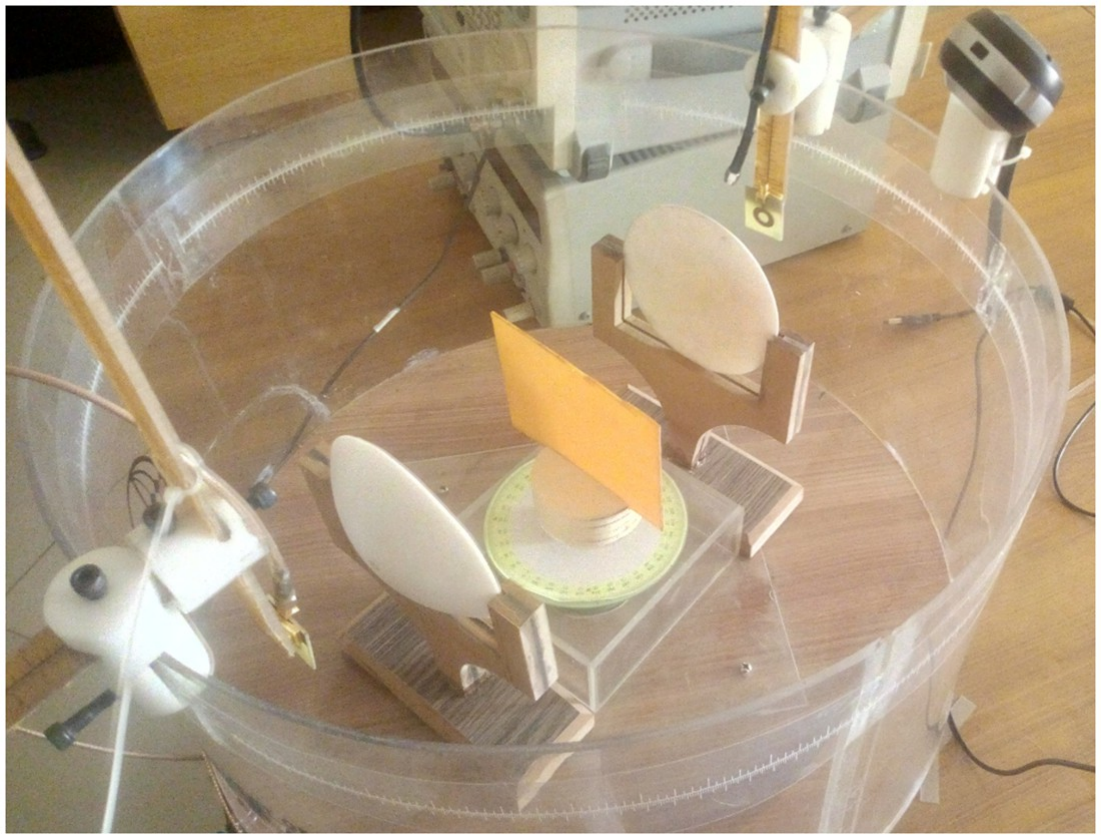
Advanced microwave hardware setup with circular ring patch antenna, lens pair, for accurate dielectric value determination”.

The utilization of circular ring patch antennas in this research paper proves their suitability for all measurements conducted, particularly due to their excellent performance in free space. By incorporating these antennas into the hardware setup, significant improvements in accuracy were achieved when calculating the dielectric value of FR4 across the entire frequency range. The addition of lenses to the setup further enhanced the performance of the patch antennas, allowing for compact and efficient beamforming capabilities. As depicted in Fig 7, this integration of lenses ensured accurate beamforming while maintaining optimal performance.

## Results and discussion

**Fig 8:**
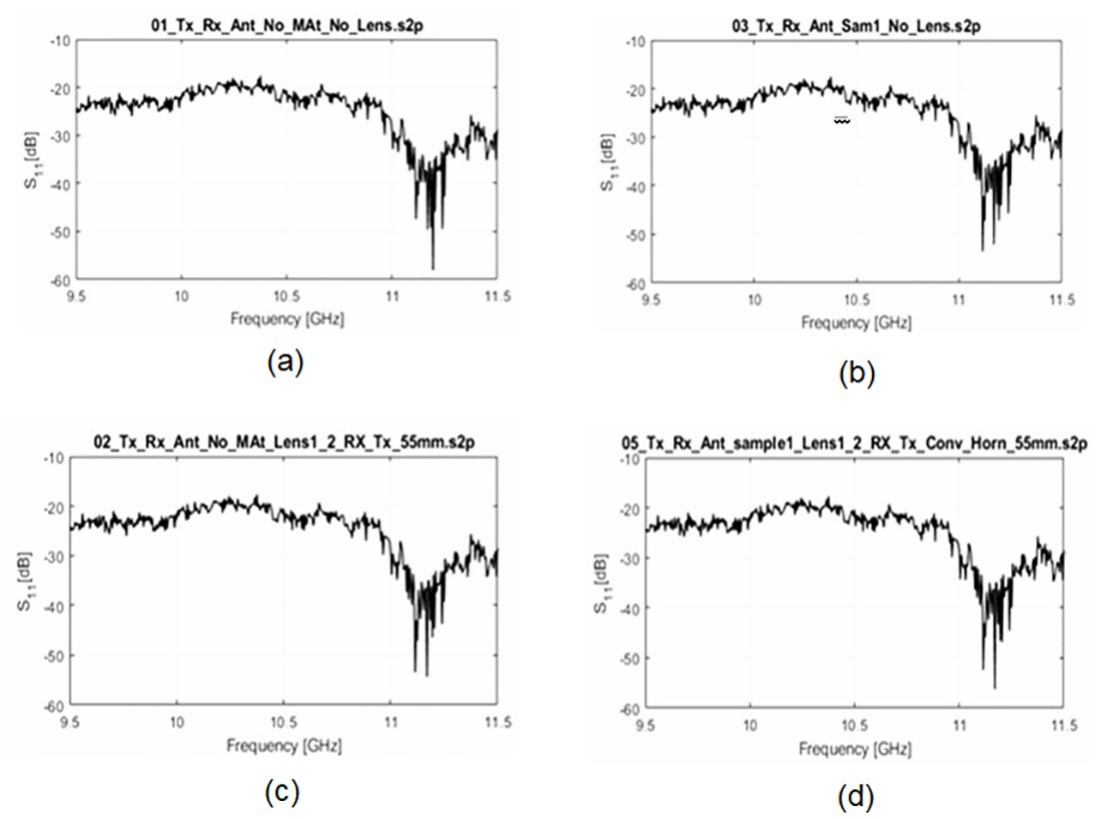
Reflection Coefficient Graphs of a Transmission System in Free Space between Transmitter and Receiver (a) Sample-Free and Lens-Free Transmission (b) Lenses-Free Transmission System with a Sample (c) A Sample-Free Transmission System with Lenses (d) Transmission System with Lenses and a Sample.

Understanding the characteristics of electromagnetic waves in transmission systems requires an understanding of the reflection coefficient, which is a key parameter. Therefore It was crucial to examine the graph of the reflection coefficient before determining the permittivity value of the FR4 material. The four reflection coefficient graphs that correspond to the various setups are obtained and have been shown in Fig 8. In the first case as shown in Fig 8(a), when the transmission system is free of lenses and FR4, is expected to result in low values for the reflection coefficients. The second scenario i.e. Fig 8(b) takes into account the use of FR4 without lenses. Depending on the material’s characteristics, single-material presence could result in frequency-dependent reflections and phase alterations. The inclusion of lenses without the use of FR4, as shown in Fig 8(c) investigates how lenses affect wave propagation and how differences in reflection coefficients can result. The fourth scenario, finally, involves both lenses and FR4 in the transmission system, which has an impact on the graph of the measured reflection coefficient as shown in Fig 8(d).

The analysis of the four reflection coefficient graphs offers important insights into how various configurations affect wave propagation in free space. It is possible to comprehend both the individual contributions of the material and lenses as well as their combined impact by contrasting and comparing the graphs. The results of this study can help with the design and improvement of transmission systems that operate in the studied frequency range.

Permittivity values were calculated within the frequency range of 8.5 GHz to 11.5 GHz. However, for the sake of convenience, Table 2 exclusively displays the values corresponding to the frequency range between 10 GHz and 11.5 GHz.

**Table 2:** Permittivity values, 10G Hz to 11.5 GHz with and without Lens.

S.No.	Frequency (GHz)	Permittivity without Lens	Permittivity with Lens
1	10	3.99	5.48
2	10.1	4.37	5.26
3	10.2	4.06	4.30
4	10.3	3.93	5.01
5	10.4	4.17	4.58
6	10.5	3.64	4.59
7	10.6	4.35	4.92
8	10.7	3.81	4.21
9	10.8	3.97	4.77
10	10.9	4.06	4.26
11	11	3.69	4.37
12	11.1	3.96	4.25
13	11.2	3.88	4.58
14	11.3	4.14	4.40
15	11.4	4.32	5.13
16	11.5	4.52	4.91
	**Average**	**4.0**	**4.7**

Table 2 presents a comparative analysis of permittivity values with and without the presence of a lens in the frequency range of 10 GHz to 11.5 GHz. Since the objective of this study is to assess the impact of a lens on the measured permittivity values and provide insights into the electromagnetic behavior of the material under investigation. By comparing the permittivity values in the ‘Permittivity without Lens’ and ‘Permittivity with Lens’ columns, it can be observed that the introduction of the lens has an effect on the measured permittivity values. The differences between the two cases demonstrate the influence of the lens on the electromagnetic properties of the material within the considered frequency range.

If we look at the table results within the range 4.26—5.48 are obtained. This corresponds to uncertainty up to 16% is observed. The incorporation of lenses yields more accurate permittivity values for FR4 materials compared to measurements conducted without lenses. On each value of the frequency band, the algorithm shows the dielectric constant value of FR4 with very little error, but by averaging the entire frequency range, we have obtained an exact constant value of FR4 with an error that is almost zero.

**Fig 9:**
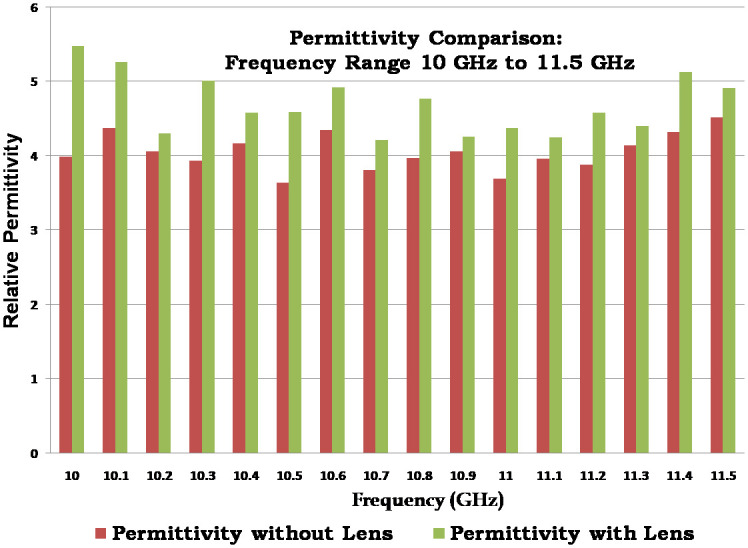
Dielectric permittivity of FR4 graph as a function of frequency ranging 10 GHz to 11.5 GHz.

Furthermore, this observed variation is visually portrayed through a bar graph, as depicted in Fig 9. The presented graph exhibits a comparative representation of the dielectric constant for the FR4 material under two distinct conditions. The red bar signifies the dielectric constant without the presence of a lens pair, while the green bar corresponds to the dielectric constant in the presence of said lens pair.

Upon careful examination of the graph, a noteworthy insight emerges. The green bar, denoting the permittivity attributed to the lens, remarkably aligns with the actual permittivity value of 4.7. This intriguing observation substantiates the notion that employing a pair of lenses yields a substantial enhancement in the accuracy of dielectric constant measurements. As a result, these refined measurements closely approximate the true value of the constant, thus offering a significant advancement in precision and reliability.

**Fig 10:**
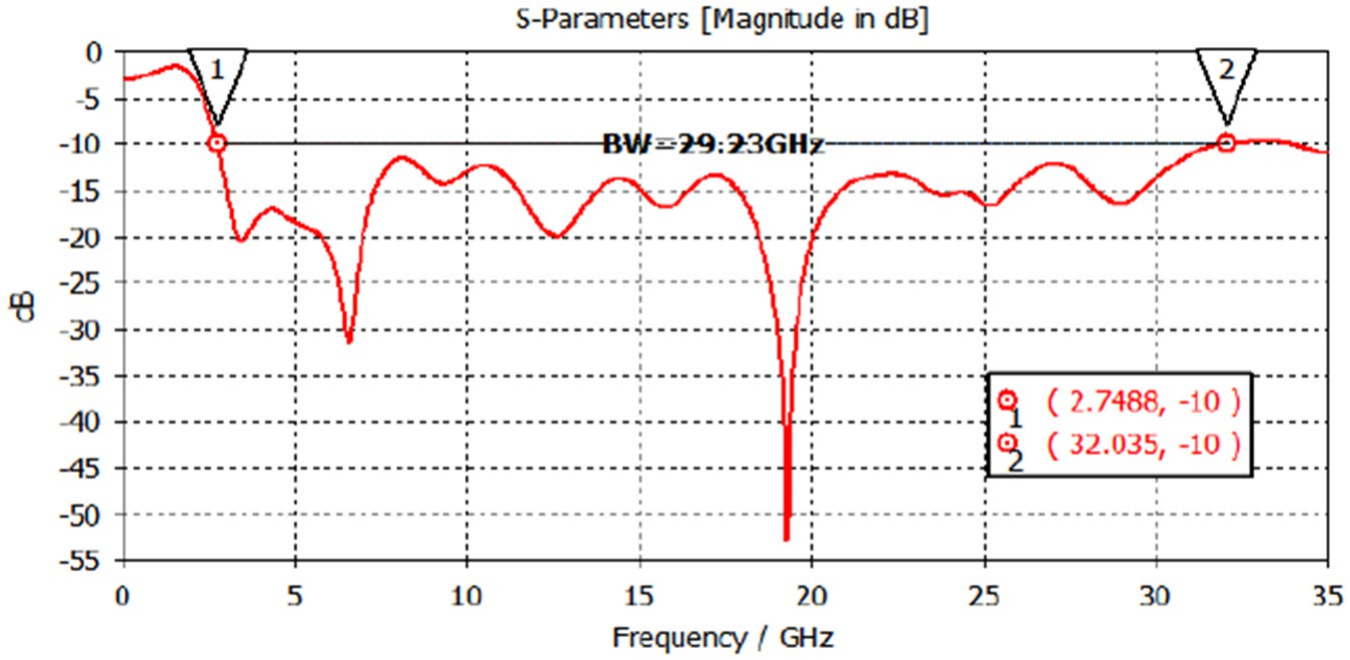
Dielectric permittivity of FR4 graph as a function of frequency ranging 10 GHz to 11.5 GHz.

The simulated reflection coefficient graph of the circular ring patch antenna is presented in Fig 10. The graph exhibits an exceptionally wide impedance bandwidth (*S*_11_ < −10 dB), spanning from 2.75 GHz to 32.035 GHz [30].

Circular ring patch antenna, offers a number of benefits, including increased bandwidth and simplicity of integration into the planned hardware. Additionally, this setup offers a chance to investigate and examine the FR4 material’s behavioural traits over a broad frequency range. Table 3 presents a comparative analysis of the proposed dielectric measurement technique with existing methods. The proposed work demonstrate improved accuracy, simplified procedures, and consistent performance across a wide range of microwave frequencies, particularly when applied to FR-4 materials.

**Table 3:** Comparison of dielectric measurement techniques.

Ref.	Published Year	Object/Material	Technique	Accuracy	Frequency of Interest	Complexity
[31]	2008	Food materials	Coaxial probe, Waveguide cell transmission line, Resonant cavity, Free space measurement of complex permittivity.	Moderate	Microwave	Moderate
[32]	2015	Breast phantom	Huygens scattering principle	High	Microwave	Moderate
[33]	2014	TLX-8, RF60A, CER10 and FR4	Microstrip transmission line method	Moderate to High	Microwave	Moderate
[34]	1990	High loss materials	Transmission/Reflection method	Moderate to High	Low to Microwave	Low
[35]	2021	FR-4	cavity resonator	High	Microwave	High
PW	2023	FR-4	Free space method	Moderate to High	Microwave	Low

PW = Present Work

## Conclusion

In conclusion, the integration of lenses and a circular ring patch antenna, as visually exemplified in Fig 6, yields a multitude of advantages that significantly enhance the overall system performance. Notably, this configuration demonstrates an amplified bandwidth while ensuring a streamlined integration process within the intended hardware framework. Moreover, this setup presents an exceptional opportunity to explore and analyze the behavioral characteristics of the FR4 material across a wide frequency spectrum.

The current endeavor has successfully ascertained the dielectric value of the FR4 material, shedding light on its intrinsic properties. Furthermore, precise dielectric values have been determined for other materials, including ceramics, Rogers, mica, among others. It is important to note that the scope of this work is limited to solid and flat materials, as stipulated by the experimental setup. However, the true essence of this endeavor lies in the unparalleled accuracy achieved throughout the research process.

Additionally, it is worth mentioning that the dimensions of the material can be effectively managed by manipulating the source placement at varying focal lengths of the lens. This technique allows for the reduction of beam width and the augmentation of directivity, further enhancing the system’s overall performance.

By leveraging the benefits of lens integration and meticulous accuracy, this research endeavor paves the way for advancements in material characterization and antenna design. The findings presented herein lay a solid foundation for future investigations in the field, opening doors to novel applications and promising avenues of research.

## Supporting information

S1 File Measurement of dielectric material properties application note. This document (application note) provides details on measuring the dielectric properties of materials using a network analyzer. It outlines methods for converting s-parameters obtained from the network analyzer measurements into usable dielectric properties, such as the permittivity of FR4. This application note served as a reference for the methods employed in our research to determine the dielectric constant of FR4 material.
(PDF)

S2 File MATLAB code for permittivity calculation using Nicholson-Ross-Weir algorithm. This MATLAB file contains code that implements the Nicholson-Ross-Weir (NRW) algorithm to calculate the permittivity (dielectric constant) of FR4 material. The code likely processes s-parameter data (potentially obtained from network analyzer measurements) to extract the permittivity value. This supporting file provides details about the specific algorithm used for permittivity calculation in our research.
(M)

S1 Table CST simulation results (table) for directivity and beamwidth after incorporating lens. This table presents the results of a CST simulation investigating the impact of a lens on the directivity and beam-width of a communication system. This data helps to understand how the lens design parameters influence the directivity and beam focusing characteristics of the communication system.
(XLSX)
