# Supplementary material for: Advancements in FR4 dielectric analysis: Free space approach and measurement validation
Source: PLoS One. 2024 Sep 12;19(9):e0305614. doi: 10.1371/journal.pone.0305614 (PMC11392535; doi:10.1371/journal.pone.0305614)
Supplement: S1 File — This document (application note) provides details on measuring the dielectric properties of materials using a network analyzer. It outlines methods for converting s-parameters obtained from the network analyzer measurements into usable dielectric properties, such as the permittivity of FR4. This application note served as a reference for the methods employed in our research to determine the dielectric constant of FR4 material. (PDF) [file pone.0305614.s001.pdf]

Measurement of Dielectric Material Properties

Application Note

Products:

- R&S®ZVA
- R&S®ZVT
- R&S®ZNB
- R&S®ZNC

The application note describes the methods to measure the dielectric properties of materials using a network analyzer. It also shows methods for converting the s-parameters to dielectric properties. Another application note will be written to show practical testing solutions with examples

ROHDE&SCHWARZ

<Application Note>

Kuek Chee Yaw

04.2012- RAC0607-0019_1_4E

Table of Contents

1 Overview 3

2 Measurement Methods 3

Transmission/Reflection Line method 5

Open ended coaxial probe method 7

Free space method 8

Resonant method 9

3 Measurement Procedure 11

4 Conversion Methods 11

Nicholson-Ross-Weir (NRW) 12

NIST Iterative 13

New non-iterative 14

Short circuit line (SCL) 15

5 Summary 16

6 Annexes 17

Annex 1: Measurement procedure 17

Annex 2: Nicholson-Ross-Weir Conversion Process 19

Annex 3: Nicholson-Ross-Weir Conversion Calculation example 22

Annex 4: NIST Iterative Conversion Method 24

Annex 5: New Non-Iterative Conversion Method 27

Annex 6: New Non-Iterative Conversion Calculation example 29

Annex 7: SCL Iterative Conversion Method 30

Annex 8: Air Gap Correction 32

7 Additional Information 34

8 Literature 34

9 Ordering Information 35

1 Overview

The measurement of complex dielectric properties of materials at radio frequency has gained increasing importance especially in the research fields, such as material science, microwave circuit design, absorber development, biological research, etc. Dielectric measurement is important because it can provide the electrical or magnetic characteristics of the materials, which proved useful in many research and development fields.

Many methods have been developed to measure these complex properties such as methods in time domain or frequency domain with one port or two ports, etc. Every method is limited to specific frequencies, materials and applications by its own constraint. With the advance of new technologies, the methods can be employed with a software program that measures the complex reflection and transmission coefficients with a vector network analyzer and converts the data into the complex dielectric property parameter.

The purpose of the application note is to describe the general procedures on dielectric material measurements using a network analyzer and to show the methods to convert from s-parameter to dielectric properties.

The application note will discuss about the four conversion methods:

- Nicolson-Ross-Weir method,
- NIST iterative method,
- New non-iterative method,
- Short circuit line method.

A process for generating an algorithm to realize the methods were presented. It is necessary to understand that the conversion methods discussed are applicable to most materials except liquids. To convert the s-parameter obtained from a liquid measurement needs a different conversion method which will be introduced only in brief in the measurement methods section.

Note that this application note only provides the basic knowledge for understanding the complex dielectric measurements and gives no practical solutions for these measurements.

2 Measurement Methods

Measurement of dielectric properties involves measurements of the complex relative permittivity (ε_r) and complex relative permeability (μ_r) of the materials. A complex dielectric permittivity consists of a real part and an imaginary part. The real part of the complex permittivity, also known as dielectric constant is a measure of the amount of energy from an external electrical field stored in the material. The imaginary part is zero for lossless materials and is also known as loss factor. It is a measure of the amount of energy loss from the material due to an external electric field. The term $\tan\delta$ is called loss tangent and it represents the ratio of the imaginary part to the real part of the complex permittivity. The loss tangent is also called by terms such as tangent loss, dissipation factor or loss factor.

The complex permeability also consists of a real part which represents the amount energy from an external magnetic field stored in the material whereas the imaginary part represents the amount of energy dissipated due to the magnetic field. Measurement on the complex permeability is only applicable to magnetic materials. Most materials are non-magnetic and thus, the permeability is very near to the permeability of free space. Table 1 shows some examples of materials with their dielectric constant and loss tangent at room temperature.

Material	Dielectric constant	Loss-tangent
Alumina	9.0	0.0006
Bacon (smoked)	2.50	0.05
Beef (frozen)	4.4	0.12
Beef (raw)	52.4	0.3302
Blood*	58	0.27
Butter (salted)	4.6	0.1304
Butter (unsalted)	2.9	0.1552
Borosilicate glass	4.3 0	0047
Concrete (dry)	4.5	0.0111
Corn oil	2.6	0.0077
Cottonseed oil	2.64	0.0682
Sandy soil (dry)	2.55	0.0062
Egg white	35.0	0.5
Fused quartz	4.0	0.0001
Fat*	5.5	0.21
Glass ceramic	6.0	0.0050
Lard	2.5	0.0360
Lung*	32	0.3
Muscle*	49	0.33
Nylon	2.4	0.0083
Olive oil	2.46	0.0610
Paper	3-4	0.0125–0.0333
Soda lime glass	6.0	0.02
Teflon	2.1	0.0003
Thermoset polyester	4.0	0.0050
Wood	1.2–5	0.0040–0.4167
*At 37°C		

Table 1 – Example on the characteristics of selected dielectric materials at room temperature and at frequency 2.45 GHz.

There are many methods developed for measuring the complex permittivity and permeability and each method is limited to specific frequencies, materials, applications and etc. by its own constraint. The application note will discuss on the following four methods:

- Transmission/reflection line method,
- Open ended coaxial probe method,
- Free space method,
- Resonant method.

Table 2 described some examples of materials, s-parameters and dielectric properties measured using various measurement methods.

Measurement techniques	Materials	S-parameters	Dielectric properties
Transmission/Reflection Line	Coaxial line, waveguides	S11, S21	ε_r, μ_r
Open-ended coaxial probe	Liquids, biological specimen, semi-solids	S11	ε_r
Free space	High temperature material, large flat solid, gas, hot liquids	S11,S21	ε_r, μ_r
Resonant Method (Cavity)	Rod shaped solid materials, waveguides, liquids	Frequencies, Q-factors	ε_r, μ_r

Table 2 – Comparison between the measurement methods.

Transmission/Reflection line method is a popular broadband measurement method. In the method, only the fundamental waveguide mode (TEM mode in coaxial line and TE mode in waveguides) is assumed to propagate.

The open-ended co-axial probe method is a non-destructive method and the method assumes only the TEM or TE mode is propagating.

The resonant method provides high accuracies and assumes the TE or TM propagation mode.

The free space method is for broadband applications and assumed only the TEM propagation mode.

Transmission/Reflection Line method

A measurement using the Transmission/Reflection line method involves placing a sample in a section of waveguide or coaxial line and measuring the two ports complex scattering parameters with a vector network analyzer (VNA). Calibration must be carried out before making the measurement. The method involves measurement of the reflected (S11) and transmitted signal (S21). The relevant scattering parameters relate closely to the complex permittivity and permeability of the material by equations. The conversion of s-parameters to complex dielectric parameter is computed by solving the equations using a program. In many cases, the method requires sample preparation such as machining so that the sample fit tightly into the waveguide or coaxial line. Calibrations in transmission line measurements use various terminations that produce different resonant behaviour in the transmission line. For good dielectric measurement, maximum electric field is required, which can be achieved by open circuited or other

capacitive termination, while calibration in coaxial line measurements can be made using either short circuited, open circuited or matched load termination. The measurement method allows the measurement of permittivity and permeability of the dielectric material.

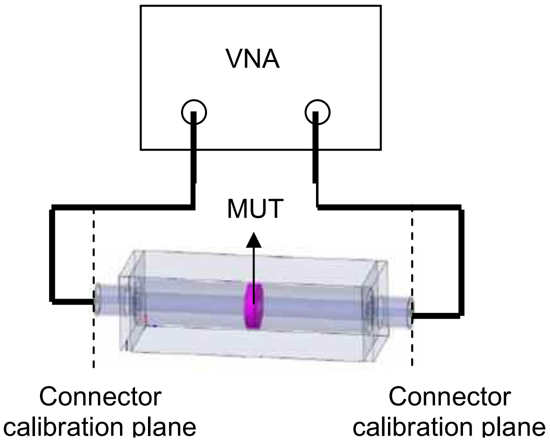

Figure 1 – Measurement using TR method with a waveguide.

The VNA is first calibrated at the connector calibration plane and the material under test (MUT) is placed in a sample holder. The MUT must fit tightly in the sample holder in order to reduce the measurement uncertainty caused by air gaps. The calibration plane can be extended to the sample surface by two methods.

The first method is to manually feed the phase factor which is equivalent to the distance between the sample surface and the connector calibration plane. The phase factor can be easily included into the measurement with the features in the VNA. The VNA will shift the calibration plane from the connector to the MUT surface.

The second method involves the de-embedding function of the VNA. The method requires measuring the s-parameter of an empty sample holder after calibration was done. The measured s-parameter of the empty holder is then input into the network analyzer. Using the de-embedding function in the VNA, the influence of the sample holder on actual material measurement can be cancelled out.

Both the methods will produce the same results. The measured s-parameters are then post processed to determine the complex dielectric properties using a program. There are various conversion methods to calculate the dielectric parameters from the measured s-parameters and the details of these methods will be explained in section “Conversion methods” in the application note.

Advantages of Transmission/Reflection line method

- Coaxial lines and waveguides are commonly used to measure samples with medium to high loss.
- It can be used to determine both the permittivity and permeability of the material under test.

Disadvantages of Transmission/Reflection line method

- Measurement accuracy is limited by the air-gap effects.
- It is limited to low accuracy when the sample length is the multiple of one-half wavelength in the material.

Open ended coaxial probe method

Open ended coaxial probe method has been used for years as a non-destructive testing method. In this method the probe is pressed against a specimen or immersed into the liquids and the reflection coefficient is measured and used to determine the permittivity.

Furthermore, for some measurements, it may not be possible to cut out the sample of a material for measurement. This is especially important in the case of biological specimens to perform in-vivo measurements because the material characteristics may change. Therefore, with this method the sample can be place in close contact with the probe without causing any changes in the material characteristics.

The reflection coefficient is measured using a vector network analyzer. The VNA with a probe system is first calibrated so that the reflection coefficient measurements are referenced to the probe aperture plane. This can be done using two methods. The first method uses reference liquids for direct calibration at the open end of the probe. It is very direct and simple. However, the uncertainties in the measurement are due to the uncertainties in the characterization of the reference liquids and the selection of reference liquids as calibration standard. In the method, all measurements are performed by placing the standards (a short, an open and a referenced liquid) at the end of the probe. The referenced liquid is used as a calibration standard and must be a liquid with “known” dielectric properties. Water, saline and methanol are usually selected as the reference liquids. Standard one port full calibration is then applied. The s-parameters measured on the MUT can be post-processed to obtain the dielectric parameters using a program.

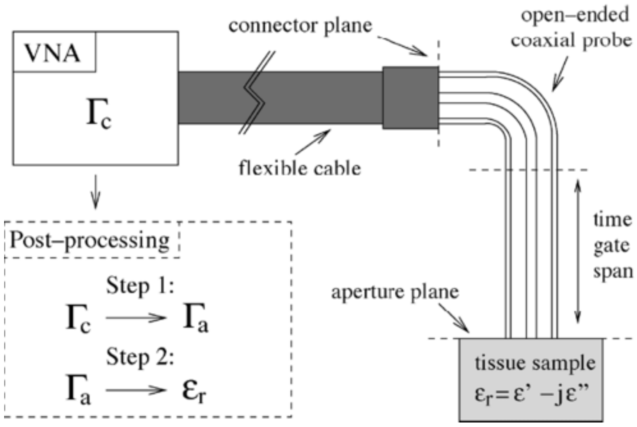

Figure 2 – Measurement of tissue sample using open coaxial probe.

Figure 2 shows the procedure to make a measurement using the second method. It uses a combination of standard calibration to calibrate at the connector plane and a simulated model of the probe to translate the connector calibration plane to probe aperture. The permittivity is then calculated from the reflection coefficient at the probe aperture. The measurement accuracy is related closely to the precision of the physical characteristics of the probe’s aperture.

The calibration process involves calibrating the VNA at the connector plane using a calibration standard (open, short and match). The probe is then connected at the connector plane. The gating function of the time domain feature in the VNA is used to

minimize the reflections from the connector. The complex coefficient data Γ_c referenced to the connector plane are recorded and post processed in two steps.

The first step involves a de-embedding model used to compensate the propagation characteristics of the probe and translate the measurement reference from the connector plane to the probe aperture plane. The model will derive the embedded reflection coefficient which is defined as Γ_a. By using the de-embedding model, the probe is treated as a two port microwave network where s-parameters are used to relate the reflection coefficient at the connector to the reflection coefficient at the aperture plane by an equation. To measure the unknown s-parameters, the measured data Γ_c and the simulated data Γ_a are used.

For the measured data, the reflection coefficients (Γ_c) of three reference liquids or samples are measured. The embedded reflection coefficient Γ_a were computed using a simulation model of an ideal probe immersed in each of the reference liquids. From the combinations of the data, the s-parameter can be determined. By determining the s-parameters, the de-embedding model can determine the unknown reflection coefficient Γ_a from the measurement reference plane reflection coefficients at $\Gamma_{c.}$

In second step, a rational function model (RFM) is applied to the Γ_a to calculate the permittivity of the sample.

Advantages of open ended coaxial probe method

- Require no machining of the sample, easy sample preparation.
- After calibration, the dielectric properties of a large number of samples can be routinely measured in a short time.
- Measurement can be performed in a temperature controlled environment.

Disadvantages of open ended coaxial probe method

- Only reflection measurement available.
- Affected by air gaps for measurement on specimen.

Free space method

Free space measurement allows measurements on MUT under high temperatures or hostile environments and generally operates in wide band frequencies. The measurement requires the MUT to be large and flat. It usually utilizes two antennas placed facing each other and the antennas are connected to a network analyzer.

Before starting the measurement, the VNA must first be calibrated. There are a number of calibration methods that can be used, such as the through-reflect-line (TRL), the through-reflect-match (TRM) and the line-reflect-line (LRL). However, the LRL calibration method can produce the highest calibration quality. The line standard can be achieved by separating the focal plane of the two antennas to approximately a quarter of wavelength. The reflect standard can be obtained by placing a metal plate on the sample holder in between the antennas.

Once calibrated, the s-parameters of an empty sample holder are measured by placing the sample holder midway between the two antennas. The MUT is then placed on the sample holder between the antennas and the s-parameter measurement is performed again. Using the de-embedding function of the VNA, the influence of the sample holder

can be cancelled out and only the s-parameter of the MUT can be determined. The s-parameter for both the reflection and transmission coefficients can be determined.

Time domain gating should also be applied to ensure there are no multiple reflections in the sample itself, though appropriate thickness should able to avoid this. It also eliminates the diffraction of energy from the edge of the antennas. The dielectric properties can be determined by post processing the measured reflection and transmission coefficient using a program.

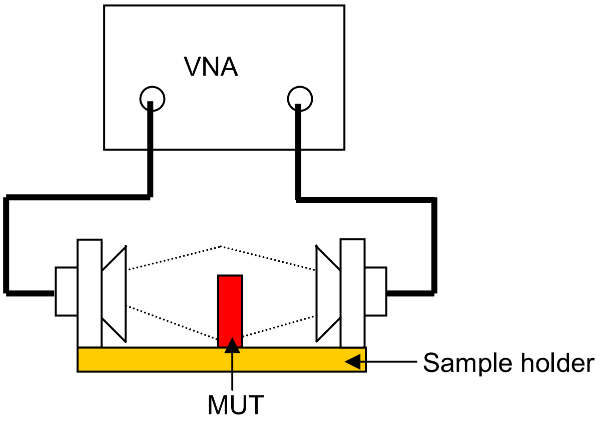

Figure 3 – Measurement of sample using free space method.

Advantages of free space method

- Can be used for high frequency measurement.
- Allows non-destructive measurement.
- Measure MUT in hostile environment.
- Both the magnetic and electric properties can be evaluated.

Disadvantages of free space method

- Need large and flat MUT.
- Multiple reflections between antenna and surface of sample.
- Diffraction effects at the edge of sample.

Resonant method

Resonant measurements are the most accurate methods of obtaining permittivity and permeability. However, there are limitations on the frequencies and loss characteristics of the materials that can be measured with the method. There are many types of resonant methods available such as reentrant cavities, split cylinder resonators, cavity resonators, Fabry-Perot resonators etc. This section will concentrate on the general overview of resonant measurements and the general procedure using a cavity resonator.

There are two types of resonant measurements commonly used. Perturbation methods are suitable for all permittivity measurements, magnetic materials and medium to high loss material measurements. Low loss measurement method is a measurement on low

loss materials using larger samples. However, the perturbation method is more popular especially using a TM cavity geometry as shown in Figure 4.
With resonance characteristics depending on the MUT in a cavity its quality factor and resonance frequency can be monitored to determine the dielectric parameters. The dielectric properties can be determined by first measuring the resonant frequency and quality factor of an empty cavity. The second step is to repeat the measurement after filling the cavity with the MUT. The permittivity or permeability of the material can then be computed using the frequency, volume and q-factor. There is no need to calibrate the network analyzer for this type of measurement.

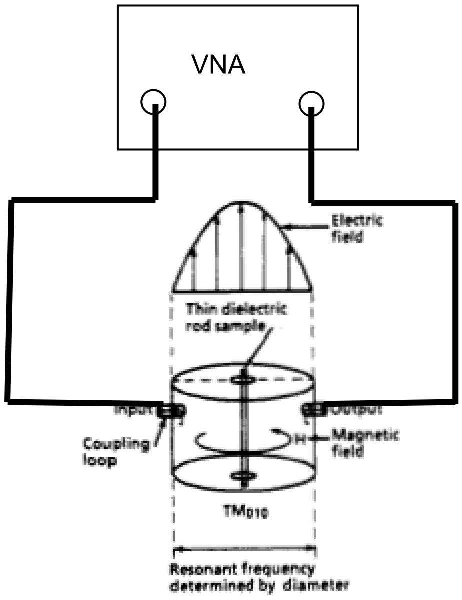

Figure 4 – Measurement of thin film using cavity resonator.

In the figure above, the rod shape solid sample is placed along the center of the cavity and the sample properties can be determined from the changes in resonant frequency and Q-factor. With the sample in the cavity any changes in the field will result in shifting of the measured resonant frequency or Q-factor.
The network analyzer needs to have a high frequency resolution in order to do this measurement. Rohde & Schwarz network analyzer have the Oven Quartz (OXCO) option that allows measurement with high frequency resolution up to 1Hz which is suitable for cavity resonant method.

Advantages of resonant method

- Ability to measure very small MUT.
- Use of approximate expression for fields in sample and cavity.

Disadvantages of resonant method

- Need high frequency resolution VNA.
- Limited to narrow band of frequencies only.

3 Measurement Procedure

The measurement setup includes a network analyzer, a software program that can be installed in the VNA or in a remote computer and the sample holder for the material under test.

The network analyzers from Rohde & Schwarz such as the ZVx series can be used for the dielectric measurement. The network analyzer has a range of calibration methods to suit different measurement methods and allows for more accurate measurements. Other features such as time domain and embedding/de-embedding functions will enhance the accuracy of the measurement result of the MUT.

A function for direct extraction of the s-parameters is available in these series of network analyzers. It is very important to have this function because it facilitates the post processing of the s-parameters using some external software programs. The external programs are then used to convert the s-parameters to the permittivity and permeability parameters.

A general procedure using the transmission/reflection method is shown in Annex 1.

4 Conversion Methods

There are various approaches for obtaining the permittivity and permeability from s-parameters. Table 3 gives an overview of the conversion methods utilizing various sets of s-parameters to determine the dielectric properties.

**Conversion techniques**	**S-parameters**	**Dielectric properties**
NRW	S11, S21, S12, S22 or S11, S21	ε_r, μ_r
NIST iterative	S11, S21, S12, S22 or S11, S21	ε_r, μ_r =1
New non-iterative	S11, S21, S12, S22 or S11, S21	ε_r, μ_r =1
SCL	S11	ε_r

Table 3 – Comparison between the conversion methods.

Each of the conversion method has different advantages and limitations. The selection of the method depends on several factors such as the measured s-parameters, sample length, the desired dielectric properties, speed of conversion and accuracies in the converted results. The application note will discuss four conversion methods. Some calculation examples are shown in Annexes.

Nicholson-Ross-Weir (NRW) method provides a direct calculation of both the permittivity and permeability from the s-parameters. It is the most commonly used method for performing such conversion. Measurement of reflection coefficient and transmission coefficient requires all four (S_{11}, S_{21}, S_{12}, S_{22}) or a pair (S_{11}, S_{21}) of s-parameters of the material under test to be measured.

However, the method diverges for low loss materials at frequencies corresponding to integer multiples of one-half wavelength in the sample which is due to the phase ambiguity. Hence, it is restricted to optimum sample thickness of $\lambda_g/4$ and used preferably for short samples.

Since there is no actual measurement, an example available was taken from a research paper [1] that shows the plot using the NRW method in determining the permittivity of a Polytetrafluoroethylene (PTFE)

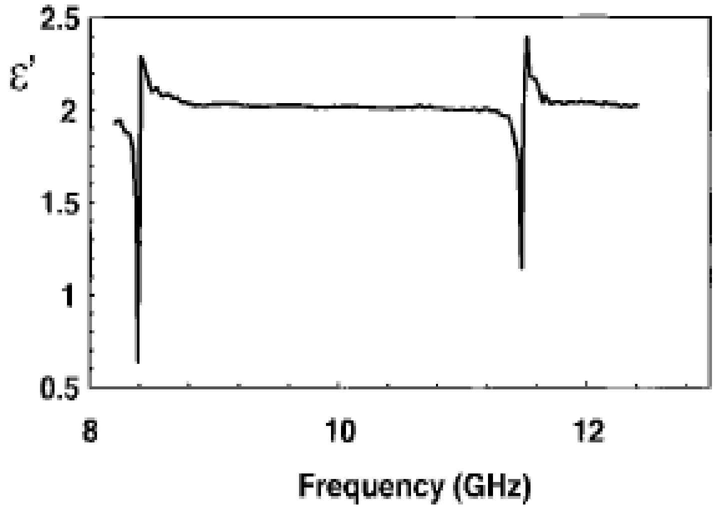

Figure 5 – Permittivity of a PTFE sample using NRW method.

From the plot above, NRW method is divergent at integral multiples one-half wavelength in the sample. This is due to the fact that at a point corresponding to the one-half wavelength the s-parameter (S_{11}) gets very small. For a small s-parameter (S_{11}) value the uncertainty in the measurement of the phase of S_{11} on the VNA is very large. Therefore the uncertainty caused the divergence at these frequencies. These divergences can be avoided by reducing the sample length, but it is difficult to determine the appropriate sample length when its ε and μ are unknown.

Advantages of NRW method

- Fast, non-iterative.
- Applicable to waveguides and coaxial line.

Disadvantages of NRW method

- Divergence at frequencies corresponding to multiples of one-half wavelength.
- Short sample should be used.
- Not suitable for low loss materials.

The procedure proposed for the NRW conversion process is shown in Annex 2. An example in performing the calculation is shown in Annex 3.

NIST Iterative method performs the calculation using a Newton-Raphson's root finding method and is suitable for permittivity calculation only. It utilizes all four (S_{11}, S_{21}, S_{12}, S_{22}) or a pair (S_{11}, S_{21}) of s-parameters of MUT to calculate the reflection and transmission coefficient. It works well if a good initial guess is available. The method bypasses the inaccuracy peaks that exist in NRW method when the sample thickness is an integer multiple of one half wavelength ($n\lambda_g/2$). It is suitable for long samples and characterizing low loss materials.

An example was taken from a technical note [2] that shows the plot using the NIST iterative method in determining the permittivity of a PTFE. This example is taken so that comparison can be made with the other methods discussed in the application note.

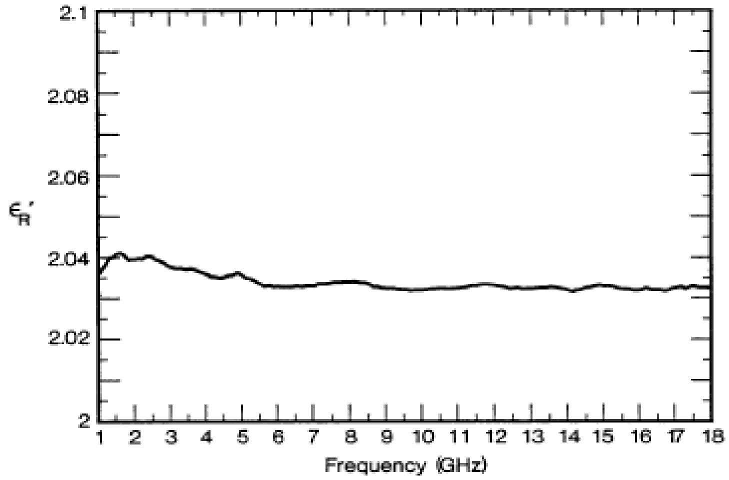

Figure 6 – Permittivity of a PTFE sample using NIST iterative method.

By using the method, a stable permittivity over the frequency spectrum can be obtained from the s-parameters as shown in Figure 6 and it allows measurements to be taken on samples of arbitrary length. The method minimizes the instability present in NRW method by setting the μ_r = 1. However, with this setting only non-magnetic materials can be measured using this method.

Advantages of NIST iterative method

- Smooth permittivity results, no divergence.
- Accurate.
- Arbitrary length of samples can be used.
- Robust for low loss and high loss materials.

Disadvantages of NIST iterative method

- Applicable for permittivity measurement only.
- Need initial guess of permittivity value.

The procedure on NIST iterative method is shown in Annex 4.

New non-iterative method is quite similar to the NRW method but with a different formulation and it is suitable for permittivity calculation for the case permeability $\mu_r = 1$. It utilizes all four (S_{11}, S_{21}, S_{12}, S_{22}) s-parameters or just two (S_{11}, S_{21}) s-parameters of MUT to calculate the reflection and transmission coefficients. The method has the advantage of being stable over a whole range of frequencies for an arbitrary sample length. The method is based on a simplified version of NRW method and no divergence is observed at frequencies corresponding to multiples of one-half wavelength in the sample. It does not need an initial estimation of permittivity and can perform the calculation very fast. The accuracies are comparable to the iterative method. The method uses a partly different formulation from the NRW method and it can be easily extended to other measuring samples, for example micro-strip or coplanar lines. It also has the permittivity and permeability appear in the expression of the effective electromagnetic parameters. The effective electromagnetic parameters represent a propagation mode.

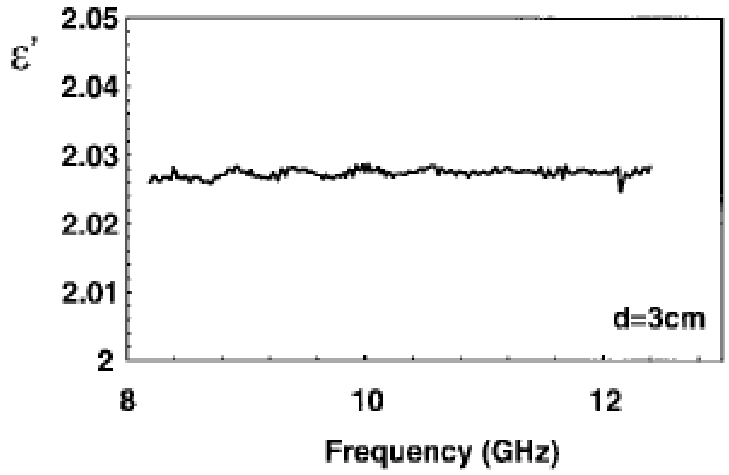

Figure 6 – Permittivity of a PTFE sample using new non iterative method.

Using an example obtained from papers [1], when comparing the method with both the NRW and NIST iterative methods as shown in Figure 6, there are no divergences observed at frequencies corresponding to integer multiples of one-half wavelength in the sample and the accuracy of the obtained permittivity is similar to the iterative method. There are no initial guesses needed and the result can be obtained very quickly.

Advantages of new non-iterative method

- Smooth permittivity results, no divergence.
- Accurate.
- Arbitrary length of samples can be used.
- Fast, non-iterative.
- No initial guess needed.

Disadvantages of new non-iterative method

- Applicable for permittivity measurement only.

The procedure for the conversion and the calculations involved are described in details in Annex 5 and 6.

Short circuit line (SCL) method is a one port measurement on coaxial lines or waveguides. It performs the calculation using the same Newton-Raphson's numerical approach as in the NIST iterative method and is suitable for permittivity calculation only. It utilizes only the S_{11} parameter of MUT to calculate the reflection coefficient. The method requires a good initial guess in order to obtain an accurate result. The method also requires the input of sample length and position for accurate measurements. The plot extracted from a technical note [2] shows the permittivity obtained when using the SCL method.

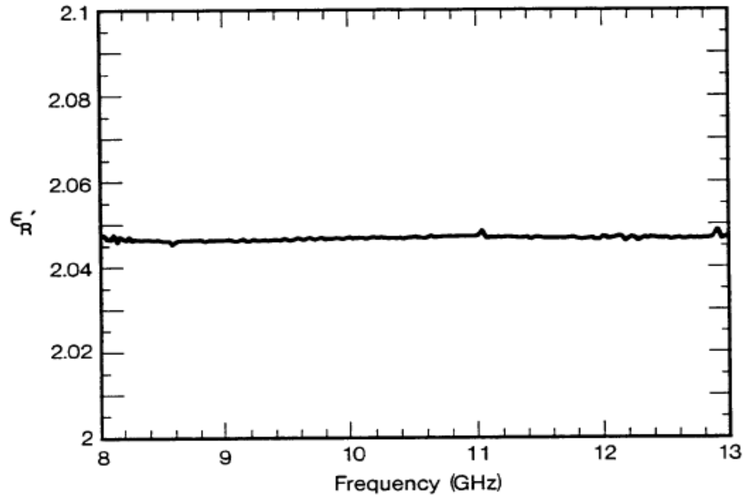

Figure 7 – Permittivity of a PTFE sample using new SCL method.

Advantages of SCL method

- Smooth permittivity results, no divergence.
- Accurate.
- Arbitrary length of samples can be used.
- For broadband measurement, preferable to use long samples for low loss materials.

Disadvantages of SCL method

- Need initial guess.
- Iterative.
- Need accurate sample length.

The procedure for the conversion is described in Annex 7.

5 Summary

In conclusion, the user needs to know the appropriate measurement and conversion methods for a material in order to measure its dielectric properties. It is necessary to use the right methods for the material to be measured because specific method is applicable to specific material. If the wrong methods are used, the measurement results will not be satisfactory. Table 4 presents a general guide of measurement and conversion methods for the different materials.

Materials / Length / magnetic properties	Measurement methods	Conversion methods	Speed	Accuracy
Lossy solids + short + non-magnetics	TR	NRW	Fast	Medium
Lossy solids + short + magnetics	TR	NRW	Fast	Medium
Low loss solids + long + non-magnetics	TR	NIST iterative	Slow	Good
Low loss solids + long + non-magnetics	TR	New non-iterative	Fast	Good
Biological specimen	Open-ended coaxial probe	RFM	Fast	Good
Liquids	Open-ended coaxial probe	RFM / Reference liquids	Fast	Good / Medium
Semi-solids	Open-ended coaxial probe	RFM	Fast	Good
High temperature solids+ large/flat+ non-magnetic	Free space	NIST iterative / New non iterative	Slow / Fast	Good
High temperature solids+ large/flat+ non-magnetic	Free space	NRW	Fast	Medium
Low loss solids+ small + magnetic	Resonant	Frequency & Q-factors	Slow	Good
Low loss solids + small + non-magnetic	Resonant	Frequency & Q-factors	Slow	Good

Table 4 – Materials with preferred measurement and conversion methods.

Besides the measurement and conversion methods, speed and accuracy are important criterias too. Speed involves how fast the measurement methods are able to extract the s-parameters and the speed of the conversion methods. Accuracy depends on the calibration methods and the conversion method utilized. Hence, this application note is primarily aimed at providing a basic knowledge in measuring dielectric properties of materials.

6 Annexes

Annex 1: Measurement procedure

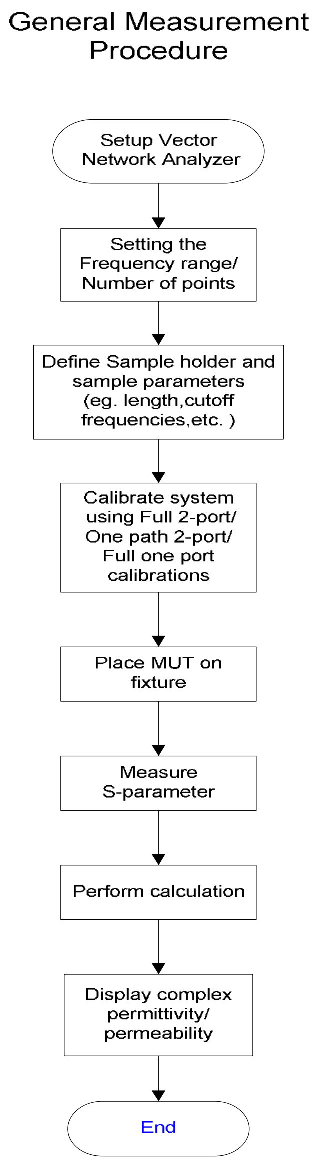

Figure 8 – A general procedure used for performing the dielectric properties measurement.

A general measurement procedure as shown in Figure 8 describes the step by step process in measuring the dielectric properties using the transmission/reflection line method. The following are descriptions on each process:

1) Setting up the network analyzer includes:
   - Cable connections to VNA and MUT or sample holder.
   - Connection between VNA and external PC.
   - Have appropriate software or driver installed in the PC or VNA.
2) Setting the following parameters in network analyzer
   - Frequency range.
   - Number of points.
3) Determine material under test and sample holder parameters
   - Sample distance.
   - Cutoff frequencies of sample holder.
   - Air gap data of MUT.
   - Sample holder dimension.
4) Calibrate the system using full two port calibration method. Prior to performing the calibration, the user must have the data of the calibration kit available and the data must be input through the calibration kit's user interface in the network analyzer.
5) If a sample holder was involved in the measurement, there are two methods to eliminate the influence of the sample holder. The first method requires the user to first calibrate without the sample holder. The electrical length of the sample holder can be determined by measuring an empty sample holder using functions such as phase or group delay. Once the electrical length is determined, it can be input to the network analyzer. The second method requires the user to use the de-embedding function available in most network analyzers to cancel out the sample holder influences.
6) The MUT can be solids, material in waveguide or coaxial transmission lines.
7) Extracts the s-parameters using functions in the network analyzer.
8) Use an external program to perform the conversion of s-parameter to dielectric properties using appropriate conversion method.

An external program used to perform the conversion of s-parameters to dielectric properties should fulfill the following requirements:

- Ability to controls and allows specific settings on the network analyzer (e.g. GPIB, LAN, RS-232, etc.).
- Allow definitions of sample and sample holder parameters (e.g. sample length, sample thickness, cutoff frequencies, sample to holder distance, etc.).
- Perform the calculations using various mathematical models (NRW model, iterative model, etc.).
- Ability to plot various type of measurements (e.g. ε', ε'', µ', µ'', loss tangent, etc.).

Annex 2: Nicholson-Ross-Weir Conversion Process

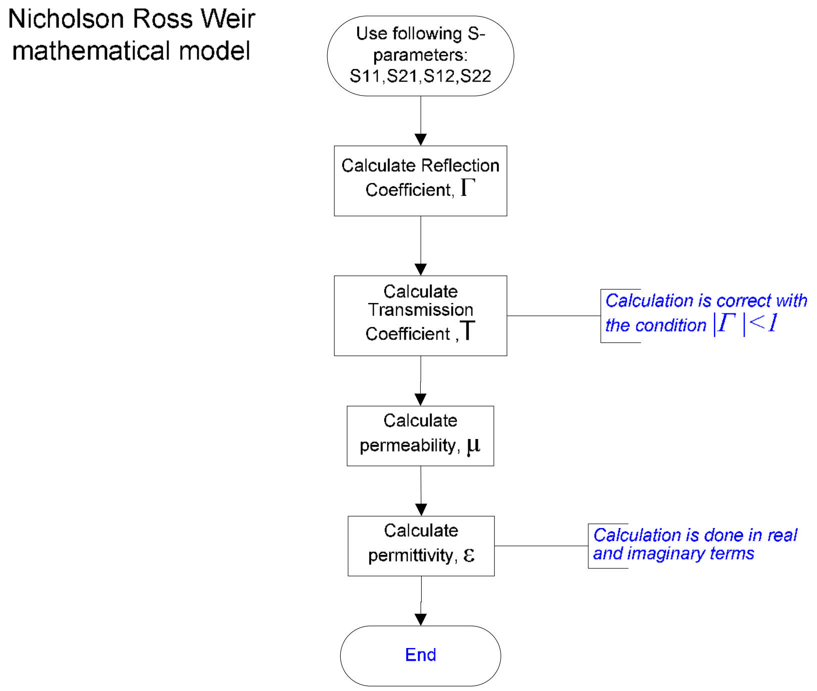

Figure 6 – The process for the NRW method.

The procedure proposed by NRW method is deduced from the following equations:

$$S_{11} = \frac{\Gamma(1-T^2)}{(1-\Gamma^2T^2)} \quad \text{and} \quad S_{21} = \frac{T(1-\Gamma^2)}{1-\Gamma^2T^2}$$

These parameters can be obtained directly from the network analyzer.

The reflection coefficient can be deduced as:

$$\Gamma = X \pm \sqrt{X^2-1} \qquad \text{---------- (1.1)}$$

where $|\Gamma_1| < 1$ is required for finding the correct root and in terms of s-parameter.

$$X = \frac{S_{11}^2 - S_{21}^2 + 1}{2S_{11}} \qquad \text{---------- (1.2)}$$

The transmission coefficient can be written as:

$$T = \frac{S_{11} + S_{21} - \Gamma}{1-(S_{11}+S_{21})\Gamma} \qquad \text{---------- (1.3)}$$

The permeability is given as:

$$\mu_r = \frac{1+\Gamma_1}{\Lambda(1-\Gamma)\sqrt{\frac{1}{\lambda_0^2}-\frac{1}{\lambda_c^2}}} \quad \text{---------- (1.4)}$$

where λ_0 is free space wavelength and λ_c is the cutoff wavelength and

$$\frac{1}{\Lambda^2} = \left(\frac{\varepsilon_r * \mu_r}{\lambda_0^2} - \frac{1}{\lambda_c^2}\right) = -\left(\frac{1}{2\pi L}\ln\left(\frac{1}{T}\right)\right)^2 \quad \text{---------- (1.5)}$$

The permittivity can be defined as

$$\varepsilon_r = \frac{\lambda_0^2}{\mu_r}\left(\frac{1}{\lambda_c^2} - \left[\frac{1}{2\pi L}\ln\left(\frac{1}{T}\right)\right]^2\right) \quad \text{---------- (1.6)}$$

Equation (1.5) and (1.6) have an infinite number of roots since the imaginary part of the term $\ln\left(\frac{1}{T}\right)$ is equal to $j(\theta + 2\pi n)$ where n= 0, $\pm$ 1, $\pm$ 2…, the integer of (L/λ_g). The n can be determined by two methods.

First method is by analysis of group delay. The equation (1.5) is ambiguous because the phase of the transmission coefficient (*T*) does not change when the length of the material is increased by a multiple of wavelength. Delay through the material is a function of the material total length and can be used to resolve this ambiguity.

The phase ambiguity can be resolved by finding a solution for ε_r and μ_r from which a comparison is made between the measured group delay and the calculated group delay to find a correct value of n.

The calculated group delay can be determined from:

$$\tau_{\text{cal}} = L\frac{d}{df}\sqrt{\frac{\varepsilon_r\mu_r f^2}{c^2} - \frac{1}{\lambda_c^2}} = \frac{1}{c^2}\frac{f\varepsilon_r\mu_r + f^2\frac{1}{2}\frac{d(\varepsilon_r\mu_r)}{df}}{\sqrt{\frac{\varepsilon_r\mu_r f^2}{c^2} - \frac{1}{\lambda_c^2}}}L \quad \text{---------- (1.7)}$$

The measure group delay is

$$\tau_{\text{meas}} = -\frac{1}{2\pi}\frac{d\phi}{df} \quad \text{---------- (1.8)}$$

which can be determined directly from the network analyzer. The calculated group delay is related to the change of the wave number k with respect to the angular frequency. The correct root, n=k, is found when

$$\tau_{\text{cal-k}} - \tau_{\text{meas}} \approx 0$$

Second method is by estimating from λ_g using initial guesses values of ε_r^* and μ_r^* for the sample. From equation (1.5), we have

$$\frac{1}{\Lambda} = j\left(\frac{\gamma}{2\pi}\right) \quad \text{---------- (1.9)} \quad \text{where } \gamma = j\frac{2\pi}{\lambda_0}\sqrt{\varepsilon_r^*\mu_r^* - \left(\frac{\lambda_0}{\lambda c}\right)^2}$$

$$\Re e\left(\frac{1}{\Lambda}\right) = \frac{1}{\lambda_g} \quad \text{---------- (1.10)}$$

By equating the equation (1.9) and (1.10), λ_g can be determined and hence the n value.

Once the n value is determined, the permittivity ε_r and μ_r can be determined.

The permittivity can also be determined from the equations for (1.4) and (1.5) which avoid determining the n values. However, this is only valid for permittivity measurement as this equation assumes $\mu_r = 1$.

From the equation (1.4),

$$\frac{1}{\Lambda} = \mu_r\frac{(1-\Gamma)}{(1+\Gamma)}\sqrt{\frac{1}{\lambda_0^2} - \frac{1}{\lambda_c^2}} \quad \text{---------- (1.11)}$$

By equating this equation with (1.5), the permittivity can be obtained

$$\frac{1}{\Lambda^2} = \mu_r^2\frac{(1-\Gamma)^2}{(1+\Gamma)^2}\left(\frac{1}{\lambda_0^2} - \frac{1}{\lambda_c^2}\right) = \left(\frac{\varepsilon_r * \mu_r}{\lambda_0^2} - \frac{1}{\lambda_c^2}\right) \quad \text{---------- (1.12)}$$

which will yield

$$\varepsilon_r = \mu_r\frac{(1-\Gamma)^2}{(1+\Gamma)^2}\left(1 - \frac{\lambda_0^2}{\lambda_c^2}\right) + \frac{\lambda_0^2}{\lambda_c^2}\frac{1}{\mu_r} \quad \text{---------- (1.13)}$$

L= material length.

ε_r= relative permittivity.

μ_r=relative permeability.

ε_r^*= initial guess permittivity.

μ_r^*=initial guess permeability.

λ_g=wavelength in sample.

γ = propagation constant of material.

c=velocity of light.

f=frequency.

Annex 3: Nicholson-Ross-Weir Conversion Calculation example

For example, using a measured S-parameter values

S_{11}= 0.856 ∠ 163.2°

S_{21}= 0.609 ∠ -140.5°

Measurement frequency f = 8GHz

Sample length = 0.4cm

Sample is placed into the H-band sample holder with cutoff frequency, f_c=5.26 GHz.

λ_0 = 3.75cm

λ_c = 5.703cm

1) Calculate X from equation (1.2)

X = -0.991 + j0.171

2) Calculate Γ from equation (1.1)

$$\Gamma = \left(-0.991 + j0.171\right) \pm \sqrt{\left(1.006\angle 170.21°\right)^2 - 1}$$

Due to the condition |Γ| < 1,

Γ = (-0.991 + j0.171)+(0.384-j0.441) = -0.607-j0.27

3) Calculate T from equation (1.3)

T = -0.912-j1.035 = 1.38 ∠ -131.4°

4) From equation (1.5) , calculate $\ln\left(\frac{1}{T}\right)$

$\ln\left(\frac{1}{T}\right)$ = ln(0.725 ∠ 2.293rad

= ln(0.725) + j(2.293 +2 π n).

n can be determined by three methods as mentioned earlier. In this case, if we use the first method whereby we assume n=0 (for demonstration purposes).

$\ln\left(\frac{1}{T}\right)$ = -0.322 + j2.293

Hence,

$$\frac{1}{\Lambda^2} = -\left(\frac{1}{2\pi L}\ln\left(\frac{1}{T}\right)\right)^2 = -\left(\frac{1}{2\pi \times 0.4}\left(-0.322 + j2.293\right)\right)^2 = -0.852\angle 195.98°$$

and $\frac{1}{\Lambda} = \pm j0.923\angle 98.0°$

Because $\Re e\left(\frac{1}{\Lambda}\right) \geq 0$, then

$$\frac{1}{\Lambda} = 0.914 + j0.128 = 0.923\angle 7.97°$$

$$\frac{(1+\Gamma)}{(1-\Gamma)} = 0.211 - j0.204 = 0.293\angle -44.03°$$

5) From equation (1.4) , the permeability can be determined

$$\mu_r = \frac{0.923\angle 7.97° \times 0.293\angle -44.03°}{\sqrt{\frac{1}{3.75^2} - \frac{1}{5.703^2}}} = 1.346\angle -36.1°$$

Therefore, μ_r = 1.08– j0.79

6) From equation (1.6), the permittivity is

$$\varepsilon_r = \frac{\left(-0.852\angle 195.98° + \frac{1}{5.703^2}\right) \times 3.75^2}{1.346\angle -36.1°} = 9.21\angle 51.6°$$

Thus, ε_r = 5.7 + j7.2

Annex 4: NIST Iterative Conversion Method

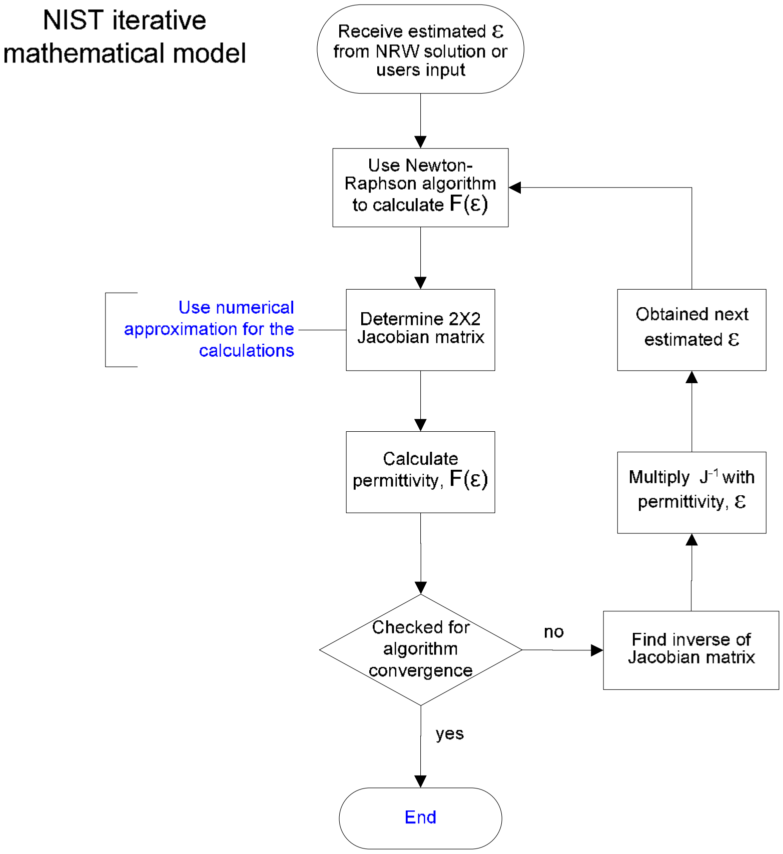

Figure 8 – The process for the NIST iterative method.

The NIST iterative method uses the NRW method to obtain the initial guess. However, if users do know the approximate permittivity value of the material, then it can be deduced from the following equations:

The reflection coefficient can be obtained as

$$\Gamma = \frac{\frac{\gamma_0}{\mu_0} - \frac{\gamma}{\mu}}{\frac{\gamma_0}{\mu_0} + \frac{\gamma}{\mu}} \quad \text{----------- (2.1)}$$

The propagation constant in air can be determined as

$$\gamma_0 = j\sqrt{\left(\frac{\omega}{c}\right)^2 - \left(\frac{2\pi}{\lambda_c}\right)^2} \quad \text{----------- (2.2)}$$

The propagation constant in material can be defined as

$$\gamma = j\sqrt{\frac{\omega^2\mu_r\varepsilon_r}{c^2} - \left(\frac{2\pi}{\lambda_c}\right)^2}$$ ---------- (2.3) where $\varepsilon = \varepsilon_0\varepsilon_r$ and $\mu = \mu_0\mu_r$.

$$c = \frac{1}{\sqrt{\varepsilon_0\mu_0}}$$ ---------- (2.4)

By replacing equation (2.4) into (2.3) will yield the following equation with μ_r =1

$$\gamma = j\sqrt{\varepsilon_r\varepsilon_0\mu_0\omega^2 - \left(\frac{2\pi}{\lambda_c}\right)^2}$$ ---------- (2.5)

From equation (2.1), the reflection coefficient can be determined with μ_r =1:

$$\Gamma = \frac{\frac{\gamma_0}{\mu_0} - \frac{\gamma}{\mu}}{\frac{\gamma_0}{\mu_0} + \frac{\gamma}{\mu}} = \frac{\gamma_0 - \gamma}{\gamma_0 + \gamma}$$ ---------- (2.6)

The transmission coefficient is

$$\mathrm{T} = e^{(-\gamma L)} = e^{-jL\left(\sqrt{\varepsilon_r\varepsilon_0\mu_0\omega^2 - \left(\frac{2\pi}{\lambda_c}\right)^2}\right)}$$ ---------- (2.7)

By solving either one of the two equations will allow the determination of the permittivity

$$F(\varepsilon_r) = S_{11}S_{22} - S_{21}S_{12} - \left[e^{-2\gamma_0(L_{air}-L)}\right]\frac{\mathrm{T}^2 - \Gamma^2}{1 - \Gamma^2\mathrm{T}^2}$$ ---------- (2.8)

$$F(\varepsilon_r) = \frac{S_{21} + S_{12}}{2\left(1 - \mathrm{T}^2\Gamma^2\right)} - \mathrm{T}\left(1 - \Gamma^2\right)e^{-j\gamma_0(L_{air}-L)}$$ ---------- (2.9)

From equation (2.8) and (2.9), the permittivity can be solved by the iterative method where the Newton numerical method for root determination is used. The algorithm is considered converged when F(ε_r)=0.

To determine the root by Newton method, a Jacobian matrix need to be computed and we can use the finite difference approximation for the matrix.

The functions for determining the permittivity can be defined as a function equivalent to equation (2.8) where

$$F(\varepsilon_{r1}) = f_1(\varepsilon', \varepsilon'')$$

$$F(\varepsilon_{r2}) = f_2(\varepsilon', \varepsilon'')$$

The Jacobian matrix is

$$J = \begin{pmatrix} \frac{f_1(\varepsilon'+h,\varepsilon'')-f_1(\varepsilon'-h,\varepsilon'')}{2h} & \frac{f_1(\varepsilon',\varepsilon''+h)-f_1(\varepsilon',\varepsilon''-h)}{2h} \\ \frac{f_2(\varepsilon'+h,\varepsilon'')-f_2(\varepsilon'-h,\varepsilon'')}{2h} & \frac{f_2(\varepsilon',\varepsilon''+h)-f_2(\varepsilon',\varepsilon''-h)}{2h} \end{pmatrix}$$ where h is small

By determining the inverse of Jacobian matrix, the small changes in permittivity function that moves the value closer to the desired value is determine as

$$\Delta\varepsilon_r = J^{-1}\varepsilon_r$$

A second iteration of this process can now be performed using the new value

$$\varepsilon_{r(new)} = \varepsilon_r + \Delta\varepsilon_r$$

The algorithm terminates once a value of ε_r is reached such that F(ε_r) is sufficiently close to zero.

The method depends on the initial guess value to yield a better approximation of the permittivity.

L= material length.

L_{air}=L_1+ L_2+L =length of air-line.

ε_r= relative permittivity.

μ_r=relative permeability.

λ_0 is free space wavelength.

λ_c is the cutoff wavelength.

c=velocity of light.

ω =angular frequency.

Annex 5: New Non-Iterative Conversion Method

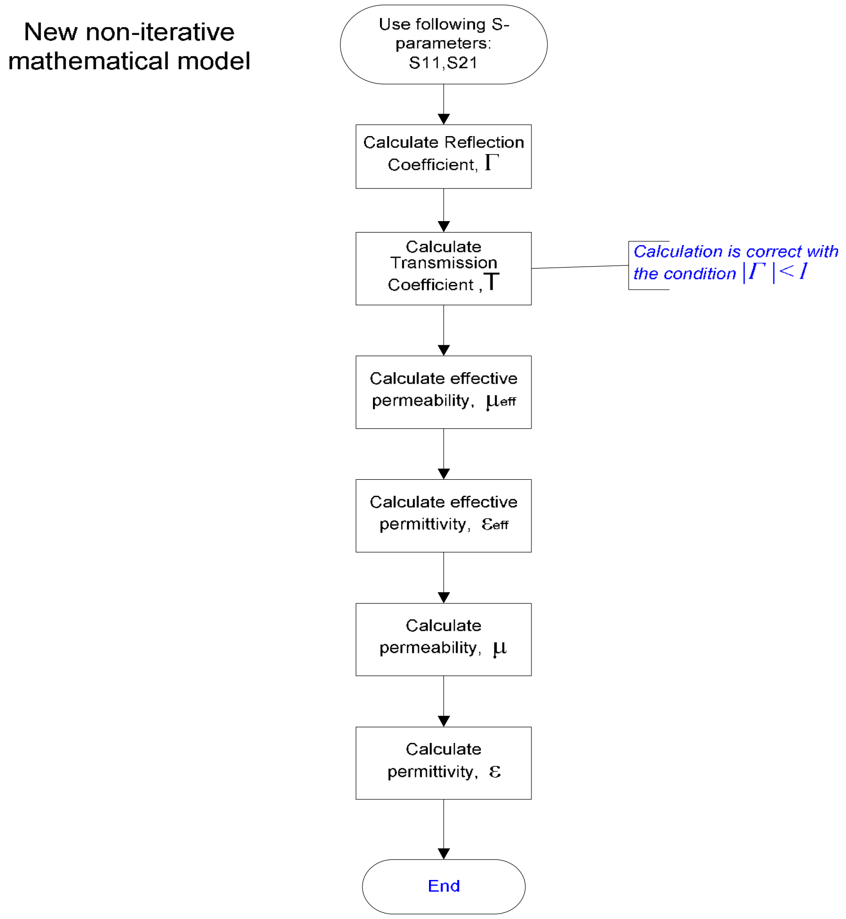

Figure 10 – The process for the new non-iterative method.

As mentioned earlier, the method is quite similar to the NRW method with the exception that it introduces the effective electromagnetic parameters and these equations are deduced as follows:

The s-parameters can be obtained directly from the network analyzer.

The reflection coefficient can be deduced as:

$\Gamma = X \pm \sqrt{X^2 - 1}$ ---------- (3.1) where $|\Gamma_1| < 1$ is required for finding the correct root.

In terms of s-parameter, it is defined as

$$X = \frac{S_{11}^2 - S_{21}^2 + 1}{2S_{11}} \quad \text{---------- (3.2)}$$

The transmission coefficient can be written as:

$$T = \frac{S_{11} + S_{21} - \Gamma}{1 - (S_{11} + S_{21})\Gamma} \quad \text{---------- (3.3)}$$

$$\frac{1}{\Lambda^2} = \left(\frac{\varepsilon_r * \mu_r}{\lambda_0^2} - \frac{1}{\lambda_c^2}\right) = -\left(\frac{1}{2\pi L}\ln\left(\frac{1}{T}\right)\right)^2 \quad \text{---------- (3.4)}$$

where λ_0 is free space wavelength and λ_c is the cutoff wavelength

With

$$\lambda_{og} = \frac{1}{\sqrt{\frac{1}{\lambda_0^2} - \frac{1}{\lambda_c^2}}} \quad \text{---------- (3.5)}$$

which represents the wavelength in empty cell

The effective electromagnetic parameters are defined as:

$$\mu_{eff} = \frac{\lambda_{og}}{\Lambda}\left(\frac{1+\Gamma}{1-\Gamma}\right) \quad \text{---------- (3.6)}$$

and

$$\varepsilon_{eff} = \frac{\lambda_{og}}{\Lambda}\left(\frac{1-\Gamma}{1+\Gamma}\right) \quad \text{---------- (3.7)}$$

With the results known from the reflection coefficient, Γ and equation (3.5), the effective parameters can be determined.

Therefore we can deduced that

$$\mu_r = \mu_{eff} = \frac{1}{\Lambda}\left(\frac{1+\Gamma}{1-\Gamma}\right)\frac{1}{\sqrt{\frac{1}{\lambda_0^2} - \frac{1}{\lambda_c^2}}} \quad \text{---------- (3.8)}$$

and

$$\varepsilon_r = \left(1 - \frac{\lambda_o^2}{\lambda_c^2}\right)\varepsilon_{eff} + \frac{\lambda_o^2}{\lambda_c^2}\frac{1}{\mu_{eff}} \quad \text{---------- (3.9)}$$

If the method is used for purely non-magnetic material where $\mu_r = \mu_{eff}$ =1, then we can express the effective complex permittivity as:

$$\varepsilon_{eff} = \varepsilon_{eff}\left(\mu_{eff}\right)^n = \left(\frac{1-\Gamma}{1+\Gamma}\right)^{n-1}\left(\frac{\lambda og}{\Lambda}\right)^{n+1} \quad \text{----------- (3.10)}$$

where n=1 for this new non-iterative method.

Annex 6: New Non-Iterative Conversion Calculation example

Using the same example as from NRW method, we can use the calculation done from step (1) to step (4).

1) From equation (3.8) , the permeability can be determined

$$\mu_{eff} = \frac{0.923\angle 7.97° \times 0.293\angle -44.03°}{\sqrt{\frac{1}{3.75^2} - \frac{1}{5.703^2}}} = 1.346\angle -36.2°$$

Therefore, μ_{eff} = 1.08– j0.79

2) The effective permittivity is calculated as

$$\varepsilon_{eff} = \frac{0.923\angle 7.97°}{\sqrt{\frac{1}{3.75^2} - \frac{1}{5.703^2}}\left(0.293\angle -44.03°\right)} = 15.68\angle 52°$$

3) The permittivity can be determined from equation (3.9)

$$\varepsilon_r = \left(1 - \frac{3.75^2}{5.703^2}\right)\left(15.68\angle 52°\right) + \frac{3.75^2}{5.703^2}\frac{1}{\left(0.99\angle -36.2°\right)} = \left(5.48 + j7.0\right) + \left(0.35 + j0.26\right)$$

Thus, ε_r = 5.7 + j7.2

The value obtained is similar to the NRW method.

Annex 7: SCL Iterative Conversion Method

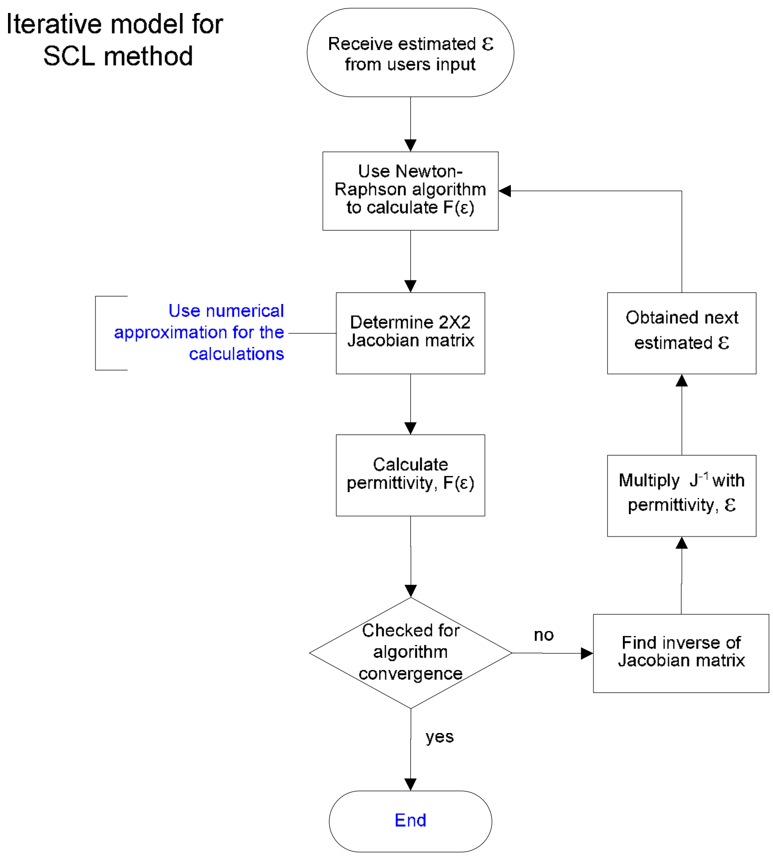

Figure 12 – The process for the SCL iterative method.

The method is similar to the NIST iterative discussed earlier with the exception that this method only need the reflection data (S11 or s22) from the network analyzer. The method is used only for permittivity measurement.

The propagation constant in air can be determined as

$$\gamma_0 = j\sqrt{\left(\frac{\omega}{c}\right)^2 - \left(\frac{2\pi}{\lambda_c}\right)^2} \quad \text{---------- (4.1)}$$

The propagation constant in material can be defined as

$$\gamma = j\sqrt{\frac{\omega^2 \mu_r \varepsilon_r}{c^2} - \left(\frac{2\pi}{\lambda_c}\right)^2} \quad \text{---------- (4.2)}$$ where $\varepsilon = \varepsilon_0 \varepsilon_r$ and $\mu = \mu_0 \mu_r$.

$$\delta = e^{-2\gamma_o L} \quad \text{---------- (4.3)}$$

$$\beta = \frac{\gamma}{\gamma_o}$$ ---------- (4.4) where 1/β is an effective impedance.

We can describe the first function of permittivity as

$$F(X1) = \tanh(\gamma L)$$ ---------- (4.5)

An equation for permittivity in terms of reflection coefficient can be determined as

$$S_{11} = \frac{-2\beta\delta + \left[(\delta+1) + (\delta-1)\beta^2\right]\tanh\gamma L}{2\beta + \left[(\delta+1) - (\delta-1)\beta^2 \tanh\gamma L\right]}$$ ---------- (4.6)

which can be re-arranged to determine the second function of permittivity

$$F(X2) = \left(s11 * \left(-2\beta + \left((\delta-1)\beta^2 - (1+\delta)X1\right) + \left(-2\beta\delta + (\delta-1)\beta^2 + (1+\delta)\right)X1\right)\right)$$ ------- (4.7)

From equation (4.7), the function allows the determination of permittivity be solved by the iterative method where the Newton numerical method for root determination is used. Please refer to the NIST iterative method for the derivation of the Jacobian matrix and the determination of the correct permittivity value through this iterative method. This method is valid only for non-magnetic materials.

This method depends on the initial guess value to yield a better approximation of the permittivity.

L = the distance from the short circuit to the sample.

ε_r = relative permittivity.

μ_r = relative permeability.

λ_c is the cutoff wavelength.

c = velocity of light.

ω = angular frequency.

Annex 8: Air Gap Correction

Once the measurement is obtained, it is necessary to correct the data taking into account the systematic uncertainties. Air gaps around the samples or imperfect waveguide wall are some of the examples. Therefore it is necessary to calculate the air gap corrections in order to obtain increased measurement accuracies for both the coaxial and waveguide samples.

ε_c = corrected permittivity values

ε_m = measured permittivity values

Coaxial gap correction calculation can be determined by first describing the dimension of the sample and coaxial holder into three terms:

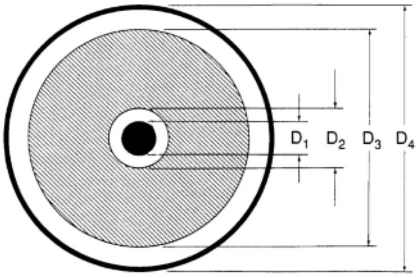

Figure 13 – Coaxial sample in holder with air gaps

$$L_1 = \ln\frac{D_2}{D_1} + \ln\frac{D_4}{D_3} \quad \text{---------- (5.1)}$$

$$L_2 = \ln\frac{D_3}{D_2} \quad \text{---------- (5.2)}$$

$$L_3 = \ln\frac{D_4}{D_1} \quad \text{---------- (5.3)}$$

With these terms, the corrected real part of the permittivity can be determined as

$$\varepsilon_c^{'} = \varepsilon_m^{'} \frac{L_2}{L_3 - \varepsilon_m^{'} L_1} \quad \text{---------- (5.4)}$$

The imaginary part of the permittivity is

$$\varepsilon_c^{''} = \left(\varepsilon_c^{'} \times \frac{\varepsilon_m^{''}}{\varepsilon_m^{'}}\right) \frac{L_3}{L_3 - L_1 \varepsilon_m^{'} \left(1 + \left[\frac{\varepsilon_m^{''}}{\varepsilon_m^{'}}\right]^2\right)} \quad \text{---------- (5.5)}$$

With the same terms, the corrected real part of the permeability is described as

$$\mu_c^{'} = \mu_m^{'} \frac{L_3 - L_1}{L_2} \quad \text{---------- (5.6)}$$

The imaginary part of the permeability is

$$\mu_c^{''} = \mu_m^{''} \frac{L_3}{L_2} \quad \text{---------- (5.7)}$$

Waveguide gap correction calculation can be determined by first describing the dimension of the sample and coaxial holder into three terms:

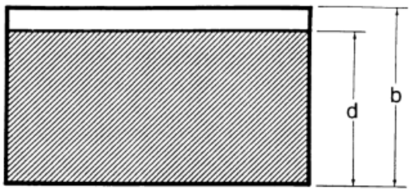

Figure 14 – Rectangular sample in holder with air gaps

d = small width of sample

b = small width of waveguide

With these terms, the corrected real part of the permittivity can be determined as

$$\varepsilon_c^{'} = \varepsilon_m^{'} \frac{d}{b-(b-d)\varepsilon_m^{'}} \quad \text{---------- (5.8)}$$

The imaginary part of the permittivity is

$$\varepsilon_c^{''} = \varepsilon_c^{'} \left(\frac{\varepsilon_m^{''}}{\varepsilon_m^{'}} \right) \frac{b}{b-(b-d)\varepsilon_m^{'}} \quad \text{---------- (5.9)}$$

As for the corrected real part of the permeability, it is described as

$$\mu_c^{'} = \mu_m^{'} \left(\frac{b}{d} \right) - \left(\frac{b-d}{d} \right) \quad \text{---------- (5.10)}$$

The imaginary part of the permeability is

$$\mu_c^{''} = \mu_m^{''} \frac{b}{d} \quad \text{---------- (5.11)}$$

7 Additional Information

Please contact your nearest Rohde-Schwarz office or customersupport@rohde-schwarz.com for additional information or further suggestions.

8 Literature

1) Abdel HakimBoughrie, Christian Legrand, Alain Chapoton, “Non-iterative stable transmission/reflection method for lowloss material complex permittivity determination”, *IEEE Transaction on microwave theory and methods, vol 45, no.1, January 1997*.

2) James Baker-Jarvis,”Transmission/Reflection and short circuit line permittivity measurements”, *Technical Note NIST.*

3) Dijana Popovic, Cynthia Beasley, Michal Okoniewski, John H.Booske,”Precision open-ended coaxial probes for in-vivo and ex vivo dielectric spectroscopy of biological tissue at microwaves frequencies”, *IEEE Transaction on microwave theory and methods, vol 53, no.5, May 2005*.

4) “Measuring the dielectric constant of solids with HP8510 network analyzer”, *Hewlett Packett application note 8510-3 August 1985.*

5) Madhan Sundaram, Yoon Kang, SM Shajedul Hasan, Mostofa K Howlader, “Measurement of Complex material properties using transmission/reflection method”, *Dept of electrical and computer engineering , university of Tennessee, Knoxville.*

6) Mohamad Nurul Afsar, James R Birch, R N Clarke, ”The measurement of the properties of materials”, *IEEE Trans. Microwave Theory Tech, vol MTT-25, June 1977*

9 Ordering Information

Designation	Type	Frequency range	Order No.
**Base Unit**			
**Vector Network Analyzer, 2 Ports, 8 GHz, N**	ZVA8	300 kHz to 8 GHz	1145.1110.08
**Vector Network Analyzer, 4 Ports, 8 GHz, N**	ZVA8	300 kHz to 8 GHz	1145.1110.10
**Vector Network Analyzer, 2 Ports, 24 GHz, 3.5 mm**	ZVA24	10 MHz to 24 GHz	1145.1110.24
**Vector Network Analyzer, 4 Ports, 24 GHz, 3.5 mm**	ZVA24	10 MHz to 24 GHz	1145.1110.26
**Vector Network Analyzer, 2 Ports, 40 GHz, 2.4 mm**	ZVA40	10 MHz to 40 GHz	1145.1110.43
**Vector Network Analyzer, 2 Ports, 40 GHz, 2.92 mm**	ZVA40	10 MHz to 40 GHz	1145.1110.40
**Vector Network Analyzer, 4 Ports, 40 GHz, 2.4 mm**	ZVA40	10 MHz to 40 GHz	1145.1110.45
**Vector Network Analyzer, 4 Ports, 40 GHz, 2.92 mm**	ZVA40	10 MHz to 40 GHz	1145.1110.42
**Vector Network Analyzer, 2 Ports, 50 GHz, 2.4 mm**	ZVA50	10 MHz to 50 GHz	1145.1110.50
**Vector Network Analyzer, 4 Ports, 50 GHz, 2.4 mm**	ZVA50	10 MHz to 50 GHz	1145.1110.52
**Vector Network Analyzer, 2 Ports, 67 GHz, 1.85 mm**	ZVA67	10 MHz to 67 GHz	1305.7002.02
**Vector Network Analyzer, 4 Ports, 67 GHz, 1.85 mm**	ZVA67	10 MHz to 67 GHz	1305.7002.04
**Vector Network Analyzer, 2 Ports, 80 GHz, 1.0 mm**	ZVA80	10 MHz to 67 GHz	1312.6750.02
**Vector Network Analyzer, 2 Ports, 80 GHz, 1.0 mm**	ZVA80	10 MHz to 67 GHz	1312.6750.03
**Vector Network Analyzer, 2 Ports, 110 GHz, 1.0 mm**	ZVA110	10 MHz to 67 GHz	1312.7004.03
**Multiport Vector Network Analyzer, 2 Ports, 8 GHz,**	ZVT8	300 kHz to 4 GHz	1300.0000.08
**Multiport Vector Network Analyzer, 2 Ports, 20 GHz**	ZVT20	10 MHz to 8 GHz	1300.0000.20
**Vector Network Analyzer, 2 Ports, 4.5GHz, N**	ZNB4	9 kHz to 4.5 GHz	1311.6010.22
**Vector Network Analyzer, 4 Ports, 4.5GHz, N**	ZNB4	9 kHz to 4.5 GHz	1311.6010.24
**Vector Network Analyzer, 2 Ports, 8.5GHz, N**	ZNB8	9 kHz to 8.5 GHz	1311.6010.42
**Vector Network Analyzer, 4 Ports, 8.5GHz, N**	ZNB8	9 kHz to 8.5 GHz	1311.6010.44
**Vector Network Analyzer, 2 Ports, 3GHz, N**	ZNC3	9 kHz to 3 GHz	1311.6004.12

About Rohde & Schwarz

Rohde & Schwarz is an independent group of companies specializing in electronics. It is a leading supplier of solutions in the fields of test and measurement, broadcasting, radiomonitoring and radiolocation, as well as secure communications. Established 75 years ago, Rohde & Schwarz has a global presence and a dedicated service network in over 70 countries. Company headquarters are in Munich, Germany.

Environmental commitment

- Energy-efficient products
- Continuous improvement in environmental sustainability
- ISO 14001-certified environmental management system

Certified Quality System
ISO 9001

Regional contact

USA & Canada
USA: 1-888-TEST-RSA (1-888-837-8772)
from outside USA: +1 410 910 7800
CustomerSupport@rohde-schwarz.com

East Asia
+65 65 13 04 88
CustomerSupport@rohde-schwarz.com

Rest of the World
+49 89 4129 137 74
CustomerSupport@rohde-schwarz.com

This application note and the supplied programs may only be used subject to the conditions of use set forth in the download area of the Rohde & Schwarz website.

R&S® is a registered trademark of Rohde & Schwarz GmbH & Co. KG. Trade names are trademarks of the owners.

Rohde & Schwarz Regional Headquarters Singapore Pte. Ltd.
10 Changi Business Park Central 2
#06-01/08
Singapore 486030
